# Shifting Defect Self-Regulation via Disordered Vacancies in Hollow Tin Perovskites

**DOI:** 10.1021/acs.chemmater.5c03101

**Published:** 2026-03-21

**Authors:** Autumn N. Peters, Persephone A. Jordano, Jennifer A. Taylor, Obadiah G. Reid, James R. Neilson

**Affiliations:** † Department of Chemistry, 3447 Colorado State University, Fort Collins, Colorado 80523, United States; ‡ Renewable and Sustainable Energy Institute, 1877 University of Colorado Boulder, Boulder, Colorado 80303, United States; § Chemistry & Nanoscience Center, National Laboratory of the Rockies, Golden, Colorado 80401, United States; ∥ School of Materials Science & Engineering, Colorado State University, Fort Collins, Colorado 80523, United States

## Abstract

Tin­(II)-based hybrid halide perovskites typically suffer from severe self-doping behavior as a result of facile oxidation of Sn­(II) to Sn­(IV), leading to high carrier densities (holes) and metallic-like conductivities that limit their applications. In this contribution, we describe how substituting the large ethylenediammonium cation for methylammonium in the intentionally defective “hollow” perovskite family, MA_1–*x*_en_*x*_Sn_1–0.7*x*_I_3–0.4*x*_ (MA = methylammonium, en = ethylenediammonium), where 0 ≤ *x* ≤ 0.38, effectively minimizes the intrinsic self-doping behavior. The use of a solvent-free, mechanochemical synthesis route further circumvents oxidative side reactions typical in solution processing, enabling more precise control and understanding of both composition and defect chemistry. Dark and time-resolved microwave conductivity measurements of these materials as a function of “*x*” reveal two regimes of conductivity suppression: at low *x* incorporation (*x* ≤ 0.15), the carrier density decreases by an order of magnitude via defect-mediated charge compensation, while higher substitution (0.15 < *x* ≤ 0.38) greatly reduces the carrier mobility. At these lower substitution levels, the observations suggest that intrinsic equilibrium tin vacancies are compensated instead by ionic defects in lieu of mobile holes. For the higher substitution levels, the less mobile carriers exhibit long recombination lifetimes, consistent with polaron-mediated transport. These findings establish a strategy for relatively low iodine chemical potential synthesis and defect-driven control of the carrier concentration in tin halide perovskites, advancing the rational discovery of dopable hybrid semiconductors.

## Graphical abstract

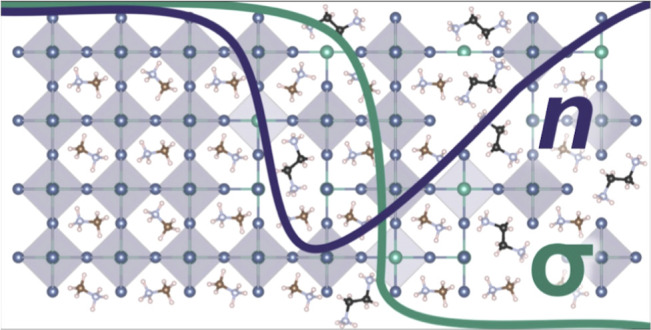

## Introduction

Hybrid organic–inorganic perovskites offer uniquely tunable structure–property relationships with applications in radiation detection,^1^ computing and thermoelectrics,^2,3^ optics and diodes,^4^ and most notably, photovoltaics (PV).^5–7^ Leading PV candidate materials primarily employ Pb­(II)-based hybrid perovskites. Materials made with Sn­(II) instead of Pb­(II) offer lower toxicity and comparable predicted optoelectronic properties.^8–12^ In practice, though, Sn­(II)-based halide perovskites typically suffer from metallic-like intrinsic carrier concentrations at equilibrium, chemically rationalized as the facile oxidation of Sn­(II) to Sn­(IV); computational studies have linked this to compensating tin vacancies.^8–17^ Furthermore, bulk Sn­(II)-based hybrid halide perovskites are traditionally synthesized using highly acidic solution precipitation susceptible to complex solution equilibria, further complicated by the light or oxygen based degradation of HI­(aq).^18^ While introduction of SnF_2_ in solution helps to mitigate this effect via the anion exchange and preferential solubilization of Sn­(IV) by fluorine,^19–21^ the electronic properties remain sensitive to the solution processing conditions. These materials are colloquially known to be “undopable” as attempting to reduce carrier concentration through traditional aliovalent substitution tends to lead to change in ionic defects rather than manipulating the carrier concentration and Fermi level.^22,23^

A class of materials known as 3D “hollow” hybrid halide perovskites is especially interesting as a platform for understanding how crystal chemistry influences defects and doping. In these materials there are two organic cations substituting on the nominally same crystallographic site: a smaller, monovalent cation (e.g., methylammonium (MA), formamidinium) and a larger, often divalent, cation (e.g., ethylenediammonium, hydroxyethylammonium),^11,24–31^ as cartooned in Figure 1. Although cations of these larger sizes typically reduce the dimensionality of hybrid perovskites (e.g., Ruddlesden–Poppper and Dion–Jacobson type), here the materials retain the 3D connectivity. The larger cation is compensated, in size and charge, by the generation of tin and iodine vacancies. In these materials, extrinsic dopant and intrinsic defects can be compensated both ionically and electronically, where ionic compensation refers to maintained charge neutrality by means of redistributing or pairing atomic point defects while electronic compensation works by means of utilizing free charge carriers (electrons and holes). These Sn­(II) vacancies are compensated ionically as opposed to electronically, which we hypothesize might help reduce, and control, high intrinsic carrier concentrations and Sn vacancies that typically arise at equilibrium and are further introduced from adventitious oxidation.^9,10,24,32^ Thus, vacancy generation via organic cation substitution offers a level of defect control that other hybrid perovskites do not offer, making these hollow structures ideal candidates for understanding how intrinsic defects can affect the electronic properties.

**Figure 1:**
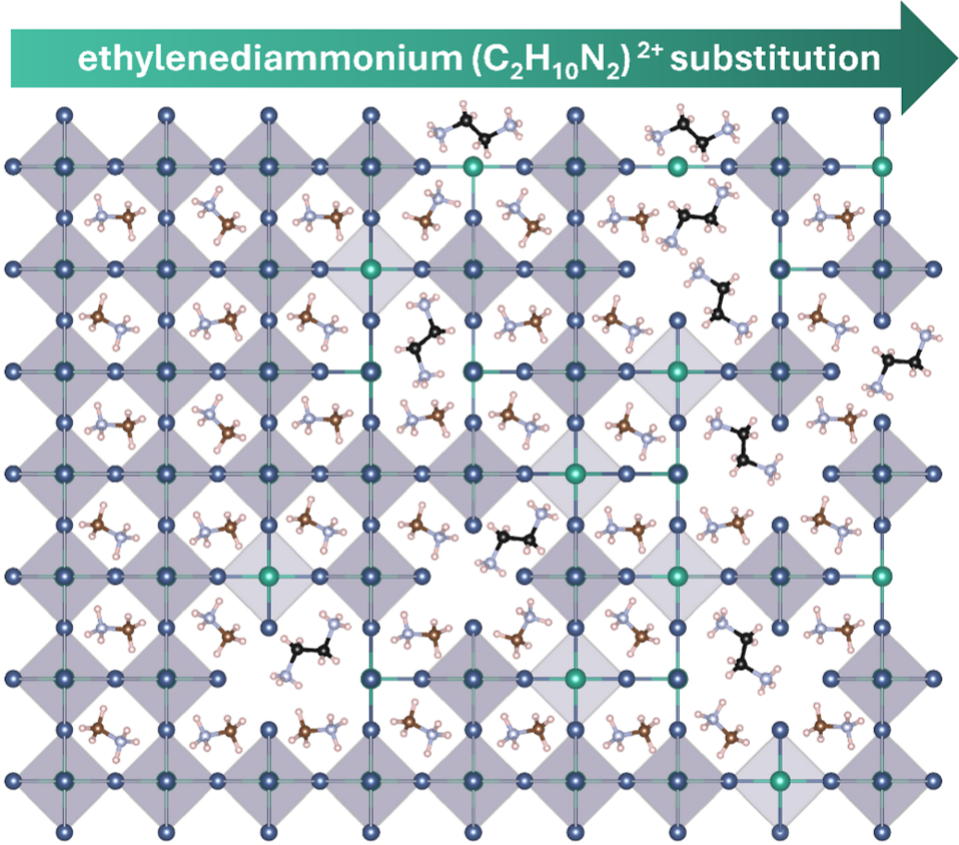
A cartoon visualization of how the crystal structure in the family MA_1–*x*_en_*x*_Sn_1–0.7*x*_I_3–0.4*x*_ 0.00 ≤ *x* ≤ 0.38 must propagate increasing amounts of Sn (green atoms) and I (purple atoms) vacancies in order to accommodate the larger size of ethylenediammonium (black carbon atoms), as compared to MA (brown carbon atoms).^33^

Nearly all synthetic methods to produce tin-iodide materials require a high iodine chemical potential, thus preventing the understanding of crystal chemical effects without direct coupling to iodine equilibria. Solution synthesis of 3D “hollow” hybrid halide perovskites yields materials with stoichiometric ratios distinct from the nominal solution ratios.^28,31,34,35^ This reflects a dynamic equilibrium dictating precipitation,^36^ thus obfuscating how to synthetically control carrier concentration, particularly in the limit of extreme iodine excess. As the preparation method of hybrid perovskites plays a crucial role in the product formation, mechanochemical synthesis has emerged as an advantageous solvent-free synthesis alternative with reproducible results.^34,37–43^ Here, solvents are avoided, opting instead for a solid-state procedure that reduces the chances of unintended equilibria impacting the family of products. In HI­(aq), iodine activity and thus chemical potential are significantly higher than in the mechanochemical approach. Higher iodine chemical potentials further promote tin vacancies (i.e., make the defect formation enthalpy lower), as is shown in computational studies.^44^

In this study, we discover how intentional introduction of ionic defects modulates the electronic carrier concentration. Discovery of a solvent-free mechanochemical synthesis method yields ideal synthesis of the 3D “hollow” perovskite family, MA_1–*x*_en_*x*_Sn_1–0.7*x*_I_3–0.4*x*_ (MA = methylammonium, en = ethylenediammonium) where 0.00 ≤ *x* ≤ 0.38. A combination of powder X-ray diffraction (PXRD) and proton nuclear magnetic resonance spectroscopy (^1^H NMR) reveals the structure and retention of the nominal synthesis stoichiometry. Optical band gaps were measured from powdered samples using UV–visible diffuse reflectance spectroscopy (UV-DRS). Densitometry data collected for powder samples validate the introduction of a significant concentration of vacancies in line with previous studies. The evolution of electronic properties (conductivity, mobility, lifetime, and carrier density) with chemical substitution is afforded by dark microwave conductivity (DMC) and time-resolved microwave conductivity (TRMC) experiments. Incorporating ethylenediammonium systematically decreases the intrinsic electronic conductivity from large, metallic values of ≥100 S/m (*x* = 0.00) to a more reasonable range for PV applications of 0.5 S/m (*x* = 0.38).^45–49^ This dramatic reduction in conductivity occurs in two qualitatively assigned regimes resulting from independent, defect sensitive experiments. First, for low *x* incorporation, the carrier density drops precipitously, consistent with our hypothesis that the intentional introduction of ionically compensated tin vacancies effectively minimizes electronic defects. Thereafter, for high *x* incorporation, the electronic mobility suffers from a high overall defect concentration. This neat synthetic pathway outlines a way to obtain bulk, highly crystalline, phase-pure, 3D “hollow” hybrid halide perovskites while highlighting the role that crystal chemistry enables the intentional control of carrier concentrations in these “undopable” materials.

## Experimental Methods

### Starting Materials

Ethylenediamine (99%, Alfa Aesar), iodine (>99.8%, Sigma-Aldrich), tin (99% granular 20 mesh, J.T. Baker Chemical Co.), stannous chloride (99%, Fischer Chemical), hydroiodic acid (stabilized with H_3_PO_2_, 57% in H_2_O, Sigma-Aldrich), hypophosphorous acid solution (50 wt % in H_2_O, Sigma-Aldrich), and methylamine solution (33 wt % in abs ethanol, Sigma-Aldrich) were purchased from the stated commercial suppliers and used without further purification.

### Synthesis of Precursors

#### Methylammonium Iodide (MAI)

Aqueous hydroiodic acid (57% w/w in H_2_O, 12 mL) and methylamine solution (33 wt % in absolute ethanol, 24 mL) were reacted and stirred in a 0 °C ice bath for 2 h. The solution was removed from the ice bath and subsequently stirred at 60 °C until the solution had evaporated, yielding the white crystalline MAI solid. The resulting solid was then washed with diethyl ether six times (40 mL/wash) via centrifugation and placed into a vacuum oven overnight at 60 °C. This is an adapted procedure from the reported ref 50. The white MAI powder was finely ground for PXRD analysis to be confirmed pure.

#### Ethylenediammonium Diiodide (enI_2_)

Aqueous hydroiodic acid (57% w/w in H_2_O, 12 mL) and an ethylenediamine solution (2.40 mL) were reacted at room temperature with an immediate precipitation of the white enI_2_ solid. The resulting solid was then washed with diethyl ether 10 times (40 mL/wash) via centrifugation and placed into a vacuum oven overnight at 60 °C. The fine, off-white enI_2_ powder was finely ground for PXRD analysis to be confirmed pure.

#### Tin­(IV) Iodide (SnI_4_)

Using a 2:1 stoichiometric ratio, solid iodine (4.052 g, 15.96 mmol) was ground into a uniform, fine powder using an agate mortar and pestle and mixed with granular (20 mesh) tin (0.9477 g, 7.983 mmol). The resulting mixture was added to a quartz tube. The tube was then briefly evacuated (<30 s to negate iodine sublimation), flame-sealed under static vacuum (∼50 mTorr), and placed into a muffle furnace. The temperature was set to 200 °C for 60 h at a heating ramp rate of 20 °C/min followed by furnace cooling to room temperature. The resulting bright orange powder was finely ground for PXRD analysis to be confirmed pure. This procedure is reported in previously published work.^51^

#### Tin­(II) Iodide (SnI_2_)

Using a 1:1 stoichiometric ratio, tin­(IV) iodide (2.52 g, 4 mmol) and granular (20 mesh) tin (0.478 g, 4 mmol) were mixed together in a mortar and pestle. Making sure the two solids were nominally homogenized by mixing, the resulting mixture was added to a quartz tube. The tube was then briefly evacuated (<30 s to negate iodine sublimation), flame-sealed under the static vacuum (∼50 mTorr), and placed into a muffle furnace. The temperature was set to 600 °C for 32 h at a ramp rate of 1.67 °C/min followed by furnace cooling to room temperature. The resulting bright orange-red, air-sensitive powder was finely ground, in an inert atmosphere (glovebox), for PXRD analysis to be confirmed pure. This procedure is reported in previously published work.^51^

### Mechanochemical Synthesis of MA_1–*x*_en_*x*_Sn_1–0.7*x*_I_3–0.4*x*_

All sample manipulations during the synthesis of MA_1–*x*_en_*x*_Sn_1–0.7*x*_I_3–0.4*x*_ where A-site % *x* = 0.00, 0.06, 0.10, 0.15, 0.25, 0.30, 0.34, and 0.38 were performed in inert atmospheres. These samples were prepared mechanochemically. In order to target the literature formula materials, MA_1–*x*_en_*x*_Sn_1–0.7*x*_I_3–0.4*x*_, salt precursors were stoichiometrically reacted in a ball mill with a 3 g batch product targeted. The values noted in the following procedure correspond to the *x* = 0.25 constituent. First, MAI (0.7075 g, 4.5 mmol) and enI_2_ (0.4690 g, 1.5 mmol) were added to a zirconia, 20 mL ball mill chamber with 3 large (7 mm) zirconia mixing balls. The chamber was sealed in an inert argon atmosphere and loaded into a Retsch CryoMill (though running at ambient temperature) for a 30 min, 30 Hz milling schedule. The chamber was brought back into an inert argon atmosphere where the tin iodide (1.8238 g, 4.9 mmol) was added to the organic mixture. The chamber was sealed and once again loaded into the ball mill for a second 90 min, 30 Hz mill with all constituents. The chamber was once again brought into an inert argon atmosphere where the now black powder was harvested and pelletized (0.25″ diameter pellets). The pellets were added to a quartz ampule that was subsequently flame-sealed under vacuum (<60 mTorr) and placed into a convection oven to anneal at 200 °C. For samples with *x* < 0.30, an 18 h anneal was sufficient to equilibrate and fully incorporate the ethylenediammonium diiodide salt. For samples with *x* > 0.30, a 36 h anneal time was required to fully incorporate the enI_2_. After annealing, samples were transferred to an argon atmosphere and finely ground for PXRD analysis to be confirmed phase pure, among other characterization. Powders where *x* < 0.30 were black in coloration while *x* > 0.30 were a dark red-black hue.

### Powder X-ray Diffraction (PXRD)

All samples were prepared on an off-axis cut zero diffraction silicon wafer. In order to extract an accurate lattice parameter, the X-ray diffraction (XRD) specimens were intimately mixed with silicon powder (1–5 mg) as an internal standard for calibrating the sample height. Measurements were completed using a Bruker D8 DaVinci powder X-ray diffractometer with Cu Kα radiation and a Lynxeye XE-T position-sensitive detector. Quantitative analysis of XRD data was performed using the Rietveld method with the TOPAS v6 software package to extract unit cell information. As the Bragg reflections broadened beyond the resolution of the instrument, the Lorentzian line width was used to estimate the crystalline microstrain during the Rietveld refinement in TOPAS v6.

### UV–Visible Diffuse Reflectance Spectroscopy (UV-DRS)

Diffuse reflectance optical spectroscopy measurements were conducted using an Ocean Optics tungsten-halogen light source (Mikropack HL-2000) and an Ocean Insight Flame Miniature Spectrometer 500 (Flame-S-VIS-NIR) equipped with an integrating sphere. A fiber-optic cable was used to illuminate the flat base of a borosilicate glass vial filled with a loose powder of the reaction products. A vial containing BaSO_4_ was used as a white reflectance standard. The Kubelka–Munk transform was calculated using the formula $\left(k/s\right)=\left({\left(1-R_{\infty }\right)}^{2}\right)/\left(2R_{\infty }\right)$ , where *k* is the absorption coefficient, *s* is the scattering coefficient, and *R*_∞_ is the diffuse reflectance. Optical band gaps are estimated from a plot of (*k*/*s*)^2^ (e.g., Tauc analysis assuming a direct gap between parabolic bands), and then by taking the tangent line of maximal slope at the absorption edge and extrapolating to the (*k*/*s*)^2^ = 0. For MASnI_3_, the band gap was unable to be extrapolated as it resided outside of the detectable region of the Si-based detector.

### Proton Nuclear Magnetic Resonance Spectroscopy (^1^H NMR)

^1^H NMR data were collected on a 400 MHz Bruker NEO400. Roughly ≈10 mg of each compound dissolved in deuterated dimethyl sulfoxide (DMSO-*d*_6_). Samples for NMR were prepared in an argon-filled glovebox and sealed with rubber septa.

### Helium Pycnometry

Experimental determination of the density was evaluated using a Micromeritics AccuPyc II 1340 helium pycnometer equipped with 0.1 and 1.0 cm^3^ aluminum sample chamber sizes. Between 500 and 600 mg of the sample (to the nearest 0.0001 g) was placed into the 1.0 cm^3^ sample cup, and sample volume determination was performed based on He displacement. Each sample was measured 10 times, and the sample volume with standard deviation was recorded. The average volume for each sample was used for the respective density calculation. Samples were not retained in a rigorously air-free environment but were transferred to the He-filled chamber in less than 2 min of air exposure.

### Dark and Time-Resolved Microwave Conductivity

DMC measurements were conducted as previously described,^52–55^ with the modification that a new experimental setup was constructed and employed at Colorado State University (CSU) to accelerate the synthesis and measurement cycle. This new system operates as a turn-key instrument for measuring the equilibrium conductivity/dielectric constant of powder samples enclosed in EPR tubes, bulk borosilicate tubes, or, in the present case, borosilicate capillaries (1.2 mm × 1.4 mm ID × OD). For these experiments, samples contained in flame-sealed, evacuated capillaries were transferred directly from an argon-filled glovebox to the vacuum line without air exposure.

A Python-based graphical user interface integrates both data acquisition and analysis using built-in lookup tables of electromagnetic simulation data that calibrate the complex conductivity (permittivity) for each kind of tube, using methods previously described.^52^ Full details are included in the Supporting Information along with a block diagram of the complete instrument (Figure S10).

TRMC measurements were conducted on established equipment^52^ with the exception that a high repetition-rate low pulse energy laser (CryLas GmbH FTSS355-Q2, 355 nm, 10 kHz, 3 μJ/pulse, 1.1 ns fwhm; 2 × 10^12^ photons/cm^2^/pulse delivered uniformly across the samples projected area) was used to excite the sample, and data were recorded on a Tektronix MSO44B oscilloscope that is capable of averaging at the full repetition rate of the laser. An average of approximately 0.5–5 M pulses were collected for each sample depending on signal strength.

The photoconductivity amplitude was calibrated as previously described,^52,54^ using simulations conducted specifically for the capillary tubes used in this work. Capillary tubes with a small ID were important because the powder conductivities for many of the material compositions reported here would have been outside the instruments dynamic range if larger tubes with more material were used. A unique sensitivity factor was calculated for each sample to account for the widely varying equilibrium conductivities and permittivities of these samples.

Photoconductivity signals are expressed as the product of the charge carrier yield (ϕ) and the sum of the electron and hole mobilities (Σμ). Each transient is fit using a well-characterized instrument response function (exponentially modified Gaussian) convolved with one or two term multiexponential function.^56,57^ The values shown in Figure 5b are obtained by summing all of the exponential prefactors, allowing us to resolve the magnitude of the yield mobility product even when the carrier lifetime is less than 10× smaller than the nominal width of the instrument response function.

## Results and Discussion

The compounds, MA_1–*x*_en_*x*_Sn_1–0.7*x*_I_3–0.4*x*_, where MA = CH_3_NH_3_^+^ and en = NH_3_(CH_2_)_2_NH_3_^2+^ were successfully prepared in bulk using a solvent-free, mechanochemical method for the nominal values, *x* = 0.00, 0.06, 0.10, 0.15, 0.25, 0.30, 0.34, and 0.38. Reactions targeting a previously reported product formula^31^ followed the balanced chemical reaction between simple salts

1 $$\left(1-x\right)MAI+\left(1-0.7x\right){SnI}_{2}+\left(x\right){enI}_{2}\to {MA}_{1-x}{en}_{x}{Sn}_{1-0.7x}I_{3-0.4x}$$

yielding nominally phase-pure cubic perovskites after understanding aspects of the synthesis process (Figure 1). Ball milling of the three salts at once yields primarily MASnI_3_, as inferred from the lattice parameter (*a* = 6.24 Å) and residual precursors (enI_2_, MAI, and SnI_2_), as observed from PXRD (Figure 2a). Upon least-squares refinement (results shown in Table S1), the MASnI_3_ peaks showed relatively low microstrain broadening. Using two milling steps, 30 min of MAI + enI_2_, followed by the addition of the SnI_2_ for another 90 min total mill, yields a product with a shifted lattice parameter (*a* = 6.27 Å) but broad diffraction peaks and residual salt impurities as observed by PXRD. These product peaks showed significant microstrain indicating the ethylenediammonium incorporating. The increased lattice parameter and peak broadening suggests that premixing the organic cations yields better incorporation of the ethylenediammonium cation into the perovskite structure, as consistent from a solution-phase precipitation synthesis.^31^ Post ball milling pelletization and annealing yield homogeneous, cubic perovskites. From samples annealed at different temperatures (100 °C, 150 °C, and 200 °C; higher temperatures yield decomposition), the PXRD patterns reveal a much sharper, shifted set of peaks (Figure 2c–e). The highest temperature, 200 °C, yields the sharpest diffraction peaks and a lattice parameter consistent with a prior study.^31^ When assessing the crystallite size of the annealed samples, values were consistent with each other, but as noted, the 100 °C showed moderate microstrain broadening which decreased for the 150 °C sample, and even further to more negligible values for the samples annealed at 200 °C.

**Figure 2:**
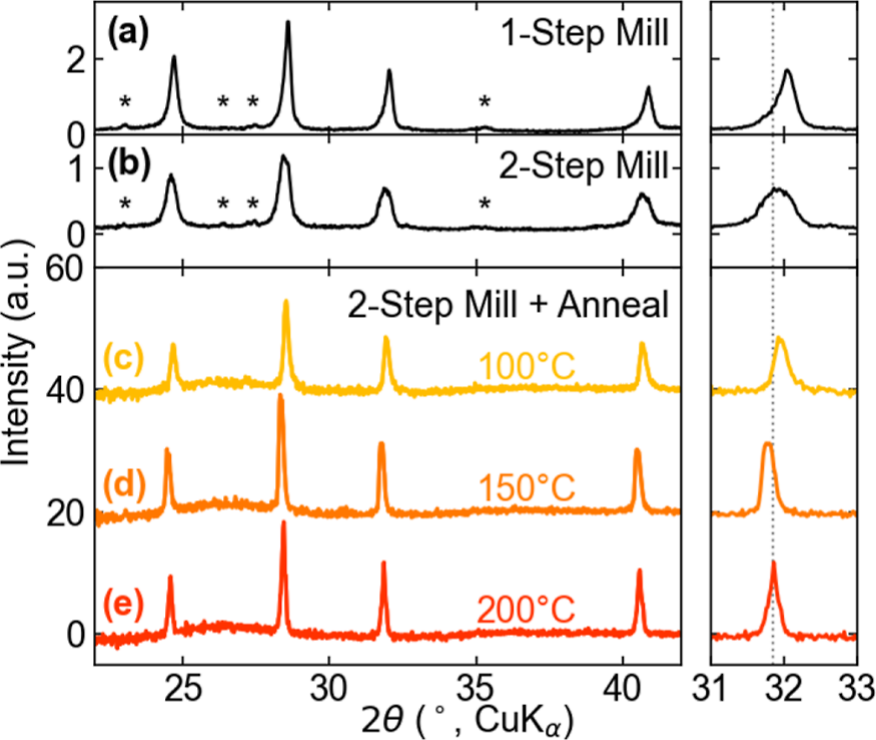
A portion of the raw PXRD patterns of the resulting products from stoichiometric reactions targeting MA_1–*x*_en_*x*_Sn_1–0.7*x*_I_3–0.4*x*_ *x* = 0.25 where (a) all salts were milled together, (b) the organic salts were milled first together and then further milled after with the inorganic salt, and where an organic and total mill were completed followed by an annealing step at (c) 100 °C, (d) 150 °C, and (e) 200 °C. A focused section of these plots (right column) highlights the peak shift and shape changes with different processing methods. The asterisks highlight impurity phase peaks seen in initial attempts.

As expected, the ratio of organic cations (MA to ethylenediammonium) input into the synthesis reaction follows through to the final crystalline products, as determined by quantitative ^1^H NMR spectroscopy. Dissolution of the perovskites in DMSO-*d*_6_ permits differentiation and quantification of the two cations by ^1^H NMR spectroscopy (Figures S1–S8).^31^ Using the methyl (−CH_3_) or ammonium (–NH3^+^) groups present in the MA cation or the methylene (−CH_2_−) and ammonium (–NH_3_^+^) groups present in ethylenediammonium, peak integration of either aprotic or protic groups validates the targeted *x* values: *x* = 0.00, 0.06, 0.10, 0.15, 0.25, 0.30, 0.34, 0.38 for MA_1–*x*_en_*x*_Sn_1–0.7*x*_I_3–0.4*x*_.

From PXRD, MA_1–*x*_en_*x*_Sn_1–0.7*x*_I_3–0.4*x*_ crystallizes as a cubic perovskite with a linearly increasing unit cell volume with *x*. The compounds are isostructural to α-MASnI_3_ (*Pm*3̅*m* space group) but show a systematic increase in unit cell dimensions with increasing *x* (Figure 3a). This manifests as a linear increase in the unit cell volume obtained from quantitative phase analysis using the Rietveld method (Figure 3b; full patterns with fits are shown in Figure S9). This expansion is consistent with insertion of a larger organic cation and/or increased Sn and I vacancy concentrations and the resulting decreased electrostatic attractions.^31^ The cubic space group and lack of superstructure peaks is evidence that the vacancies are disordered.^27,58^ While some microstrain is expected from the creation of significant vacancy content, the microstrain extracted from the quantitative phase analysis of the PXRD does not depend on *x* (Figure 3c). Together, this suggests solid-solution-like behavior with increasing ethylenediammonium content and the formation of homogeneous crystals through the solvent-free, mechanochemical synthesis after annealing.

**Figure 3:**
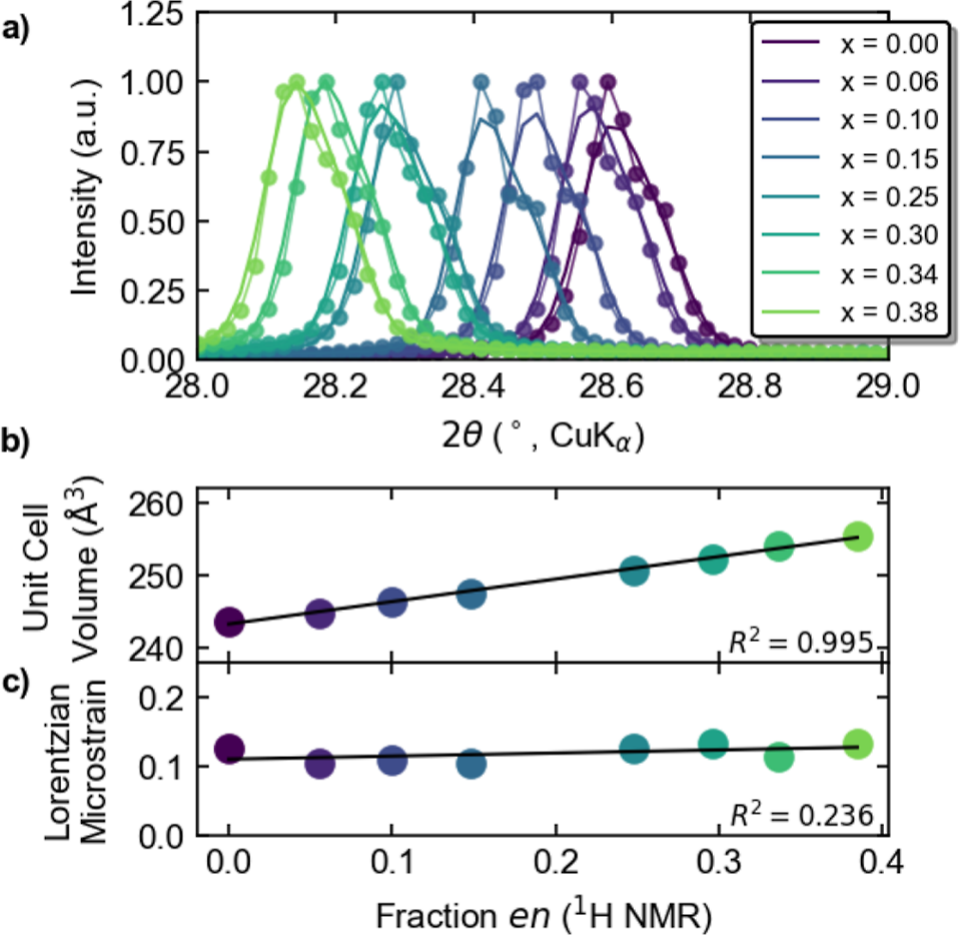
Selected region of the analyzed PXRD patterns of MA_1–*x*_en_*x*_Sn_1–0.7*x*_I_3–0.4*x*_, where 0 ≤ *x* ≤ 0.38. Plot (a) shows the raw data points as dots while the lines correspond to the full data Rietveld refinement exhibiting the shift to lower 2θ as (b) the unit cell increases in size when more ethylenediammonium is incorporated. (c) The incorporation of ethylenediammonium does not influence the PXRD-derived microstrain broadening (Table S2 for more details).

Measurement of the optical absorption confirms the reported visible light absorption and expected band gap shift expected across the values of *x* incorporation. UV–visible diffuse reflectance spectra of powder samples exhibit onset of light absorption. Fitted tangent lines to the pseudoabsorbance for a direct band gap ((*k*/*s*)^2^) extrapolated to zero absorbance are interpreted as the optical band gap (Figure 4a). The MASnI_3_ sample’s band gap was below that of our detector’s limit. The trend in band gaps shift qualitatively compares well with the previously reported trend.^31^ The presence of tailing below the extrapolated band gaps suggests the presence of optically detectable defect states,^12,59,60^ as might be expected from the presence of significant vacancy defects. The band gap of these materials increases as a function of *x*, as attributed to the decrease in orbital overlap for the atoms primarily responsible for the electronic band structure, Sn and I.^31^ This is likely most attributed to the increased lattice parameter with *x* from the decreased cohesive energy associated with increasing vacancy content; however, it could also result from direct removal of those atomic orbitals that comprise the crystal orbital. Measured band gaps manifest in a more consistently linear trend with higher band gaps than reported by Spanopoulos et al. for solution-made samples. We hypothesize this may be related to a subtle difference in local order or domain behavior as related to the different synthesis methods.^61^

**Figure 4:**
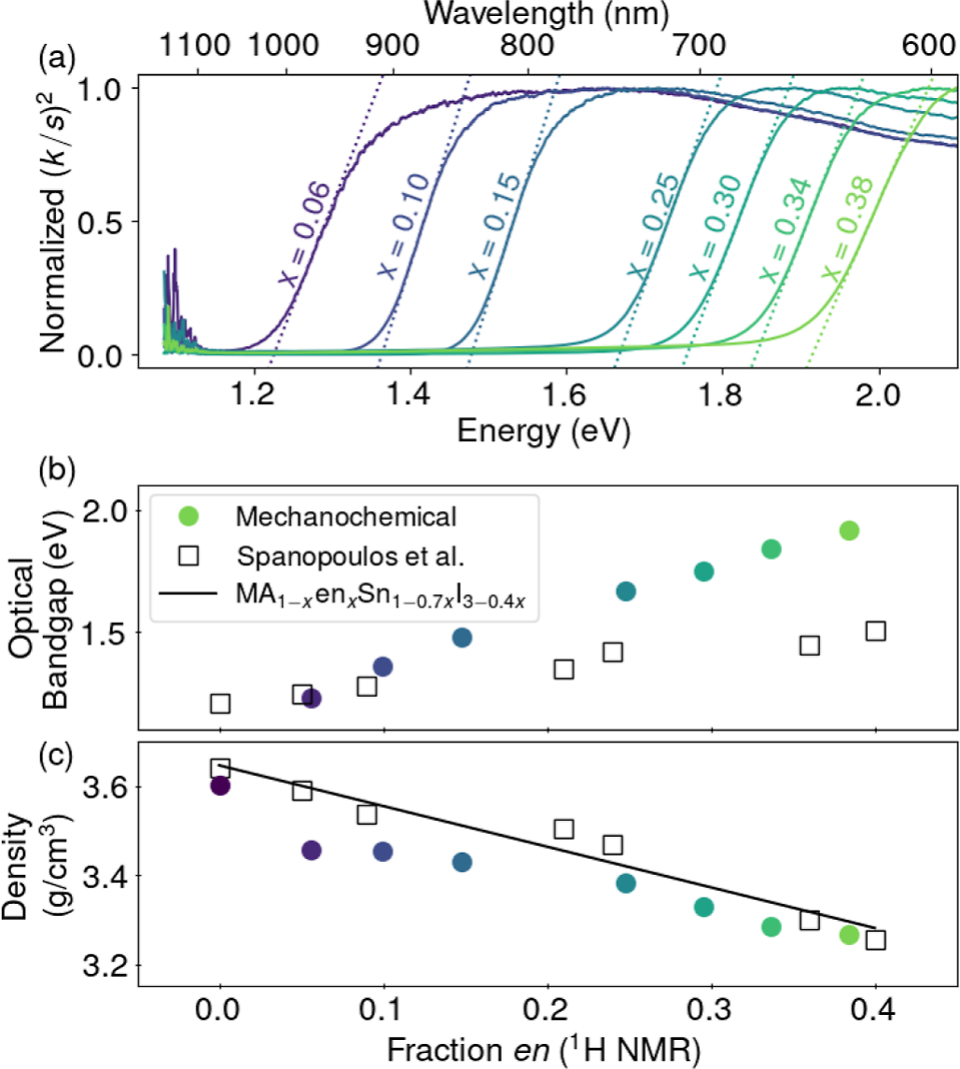
(a) Diffuse reflectance spectra shown as (*k*/*s*)^2^ collected from MA_1–*x*_en_*x*_Sn_1–0.7*x*_I_3–0.4*x*_. (b) The extracted optical band gap value highlights the linear increases in optical gap energy with incorporated fraction of ethylenediammonium. Literature values shown for comparison as open squares.^31^ (c) Experimental densities show the expected trend from the chemical formula, also in agreement with a prior study (open squares).^31^

The heavy atom Sn and I vacancies generated to accommodate the ethylenediammonium subsequently reduced the bulk density of the material. Experimental measurement of the bulk density using helium pycnometry reveals a decreasing density with increasing *x*, decreasing from 3.60 g/cm^3^ (*x* = 0.00) to 3.27 g/cm^3^ (*x* = 0.38) (Figure 4c), agreeing with literature precedent.^31^ Here, while the trend is monotonic, it is not linear and plateaus at two values of reduced density (≈3.4 and 3.27 g/cm^3^), suggesting two regimes of vacancy incorporation, as discussed later. The density is consistently lower than that of a theoretical calculation (solid line, Figure 4c) using the assumed formula weight from MA_1–*x*_en_*x*_Sn_1–0.7*x*_I_3–0.4*x*_ normalized by a linear expression for the unit cell volume with *x* (solid line, Figure 3b). We hypothesize that the literature stoichiometric formula may not fully capture the exact nature (or exact quantity) of the defects formed, especially at higher incorporation, and thus appear as larger expected densities than their realized experimental samples.

We employ DMC and TRMC experiments to assess the impact of composition on equilibrium conductivity, carrier mobility, photoexcited carrier lifetime, and carrier density. Across all compositions, the equilibrium conductivity decreases with substitution (Figure 5a). This trend was also reported but to a lesser extent in the lead-based congener, (MA)_1–*x*_(en)_*x*_(Pb)_1–0.7*x*_(I)_3–0.4*x*_.^64^ Here, we assign changes in conductivity to changes in both the mobility and carrier density. Mobility is obtained from TRMC measurements (assuming Φ = 1) which then allows us to calculate the carrier density from the equilibrium conductivity via σ = enμ. Together, in conjunction with density measurements, these reveal two apparent compositional regimes of electronic behavior: (i) *x* ≤ 0.15 yields a reduction in carrier density with little change in mobility; (ii) *x* > 0.15 exhibits a fast decline in mobility, longer photogenerated carrier lifetimes, and ultimately an apparent increase in calculated carrier density (Figure 5). While the relative dielectric permittivity also follows these trends, it remains high (≈20) even at *x* = 0.38 (Figure S11). For MASnI_3_, the microwave power reflectance revealed a conductivity higher than what can be quantified using the microwave-based measurement in our present configuration (σ ≈ 100 S/m) (Figures S14–S19). As such, we present the metallic-like minimum bound to the conductivity, which agrees well with literature values measured on optimized thin films and single crystals.^45–49^

**Figure 5:**
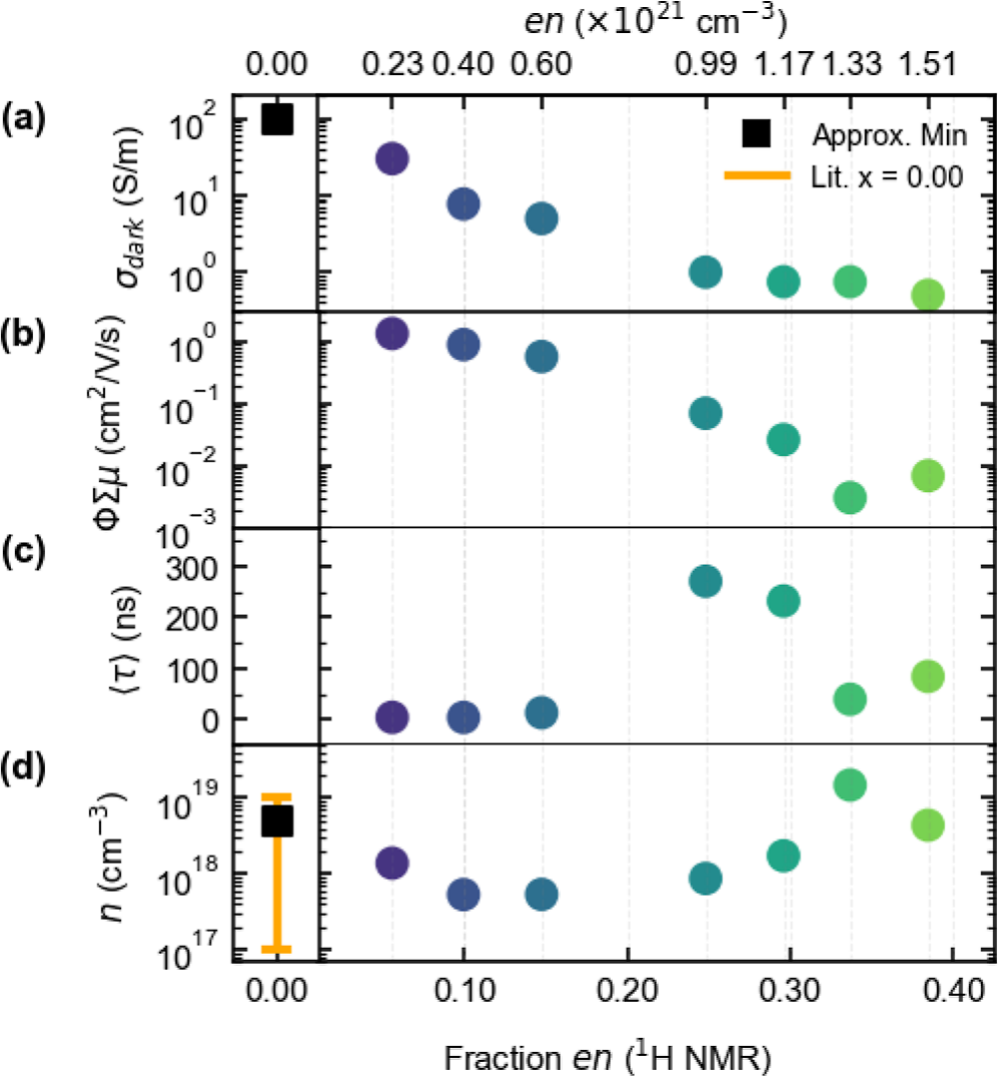
Analysis of DMC and TRMC measurements showing the (a) the dark conductivity (σ_dark_), (b) yield mobility product (ϕΣμ), (c) average lifetime of charge carriers (⟨τ⟩), and (d) the calculated carrier density (*n*) as a function of ethylenediammonium incorporation across MA_1–*x*_en_*x*_Sn_1–0.7*x*_I_3–0.4*x*_. Included on (a,d) are the approximated minimum-bound to the conductivity and carrier density for MASnI_3_ along with a range of literature-reported carrier densities.^45,46,59,62,63^ Associated error bars are smaller than the presented marker sizes (Tables S3–S5 for more details).

For low *x*, the substitution is most consistent with shifting of the defect equilibria governing charge carriers. Increasing *x* from 0.06 to 0.15 yields only a ≈2-fold decrease in mobility (1.34–0.57 cm^2^/(V s)). Across the same compositional trend, the carrier lifetime increases modestly from 4.07 ns (*x* = 0.06) to 13.21 ns (*x* = 0.15). This may be attributed to reduced pseudo first-order recombination from reduction in the dark carrier concentration. The most significant finding is the rapid decrease in carrier density from over 5.02 × 10^18^ cm^–3^ (*x* = 0.00, estimated minimum using neighboring member mobility) to 5.36 × 10^17^ cm^–3^ (*x* = 0.15).

We hypothesize that this drop in carrier density is, in part, attributable to a shift in the intrinsic defect equilibria reflecting coupling between ethylenediammonium, vacancies, and carriers that may play roles in explaining these trends. We pose that the following equilibria are an important piece of unraveling what dictates electronic properties in Sn-based perovskites. Computational studies uniformly agree that tin vacancies, intrinsic and those generated by the adventitious oxidation, are the primary compensating defect in tin halide-based perovskites (eq 2).^9,16,17,20,21,44,65–68^ Furthermore, when ethylenediammonium is introduced into the MASnI_3_ crystal, it must generate tin and iodine vacancies in order for it to sit on a MA site (eq 3).

2 $$nil⇌{v_{Sn}}^{\prime \prime }+2h^{•}$$

3 $${enI}_{2}\left(s\right)⇌{v_{Sn}}^{\prime \prime }+{v_{I}}^{•}+{{en}_{MA}}^{•}$$

The coupling of these equilibria (with the first one switched in direction) yields the net effect

4 $${v_{Sn}}^{\prime \prime }+2h^{•}+{enI}_{2}\left(s\right)⇌{v_{Sn}}^{\prime \prime }+{v_{I}}^{•}+{{en}_{MA}}^{•}$$

then reconciling species that appear on both sides leaves eq 5

5 $${enI}_{2}\left(s\right)+2h^{•}⇌{v_{I}}^{•}+{{en}_{MA}}^{•}$$

Here, nil refers to the unperturbed MASnI_3_, *v*_Sn_″ refers to a vacancy on a tin site carrying an effective 2^–^ charge, 2h^•^ denotes two electronic holes with an effective 1^+^ charge each, *v*_I_^•^ refers to a vacancy on an iodine site carrying an effective 1^+^ charge, and finally en_MA_^•^ refers to ethylenediammonium occupying a MA site and introducing an effective 1^+^ charge. We can see then in eq 5 that ethylenediammonium can be used to shift and reduce the intrinsic carrier concentration, (*h*). The nature of ethylenediammonium incorporation also lends itself to having less available Sn­(II) sites in the final structure further reducing the opportunity for Sn­(II) to unfavorably oxidize to Sn­(IV) and generate holes. This therefore helps to explain why the incorporation of small diammonium cations improves the open-circuit voltage in photovoltaic devices.^24,25,27^ Previous work on the Pb­(II)-based congeners with ethylenediammonium substitution has also alluded to the fact that small changes in preparation conditions can lead to variations in defects and overall structure influencing conductivity drastically.^64,69^ This crystal-chemical approach is thus less sensitive to the synthesis and processing conditions (e.g., HI decomposition^18^ or SnF_2_ use^19–21^ ).

The distinction, and inflection point, between the two regimes is currently phenomenological and is supported by multiple experimental observables in both density and microwave measurements. For substitution beyond the apparent threshold for regime 1, around *x* = 0.15, the conductivity continues to decrease but primarily due to a precipitous drop in carrier mobility. The extremely low mobility could arise from the extreme disruption of electronic bandwidth,^31^ defect scattering, or polaron localization. With the highly deformable Sn–I substructure, introducing a significant fraction of vacancies permits relaxation of the structure around local charge imbalances. The large increase in carrier lifetime from *x* = 0.15 to 0.25 (13.21–271.2 ns) further supports the notion of charge localization. With the increasing band gap upon substitution (Figure 4), formerly shallow acceptor states may move deeper into the gap, thus promoting localization. Meanwhile, the inferred carrier density inflects and increases for *x* > 0.15. This could reflect a change in the dominant defect equilibria. However, it is important to note the possibility that dielectric loss plays a role in the higher *x* samples as dielectric loss is indistinguishable from conductivity.^52^ If structural distortions available only at high substitution levels allow for more dipolar relaxation and phonon scattering, their relative microwave absorption can increase, leading to higher dielectric loss.

## Conclusion

Together, these findings indicate how ethylenediammonium substitution influences the intrinsic defect equilibria and its impact on the electronic conductivity, mobility, and carrier density. Discovery of a neat (solvent free), mechanochemical synthesis approach permits the synthesis of “hollow” MA_1–*x*_en_*x*_Sn_1–0.7*x*_I_3–0.4*x*_ without complicating factors related to the solution equilibria (e.g., HI decomposition) and in conditions with a lower iodine chemical potential.^18^ Furthermore, the powders produced by this method are crystallographically and electronically homogeneous. Substitution with ethylenediammonium manifests in two observable regimes of influence over the electronic properties. For *x* ≤ 0.15, the electronic properties resemble that of MASnI_3_ with a shifted intrinsic defect equilibria resulting in significantly less p-type self-doping. For *x* > 0.15 near and above, the behavior more closely resembles that of a distinct, highly defective material with more polaronic behavior with low carrier mobilities but long excited-state carrier lifetimes. Thus, the introduction of small amounts of vacancy-promoting organic cations uses crystal chemistry to minimize the high intrinsic carrier concentration and resulting conductivity that have limited the utility of materials such as MASnI_3_.

## Supplementary Material



## Notes

Relative cation ratio determination procedure and proton nuclear magnetic resonance (^1^H NMR) spectra for MA_1–*x*_en_*x*_Sn_1–0.7*x*_I_3–0.4*x*_ samples (*x* = 0.00–0.38); PXRD patterns and Rietveld refinement results for MA_1–*x*_en_*x*_Sn_1–0.7*x*_I_3–0.4*x*_ samples (*x* = 0.00–0.38) as well as synthesis of development samples; DMC system diagrams and explanation, sample resonance curves, uncertainty propagation explanation, raw and packing-fraction-corrected dielectric properties for MA_1–*x*_en_*x*_Sn_1–0.7*x*_I_3–0.4*x*_ samples (*x* = 0.06–0.38); and TRMC raw and fitted mobility-yield products for MA_1–*x*_en_*x*_Sn_1–0.7*x*_I_3–0.4*x*_ samples (*x* = 0.06–0.38) (PDF)
