# Supplementary material for: Shifting Defect Self-Regulation via Disordered Vacancies in Hollow Tin Perovskites
Source: Chem Mater. 2026 Mar 21;38(7):3374–82. doi: 10.1021/acs.chemmater.5c03101 (PMC13089876; doi:10.1021/acs.chemmater.5c03101)
Supplement: Supplementary file 1 [file cm5c03101_si_001.pdf]

Supplemental Information: Shifting Defect Self-Regulation via Disordered Vacancies in Hollow Tin Perovskites

Autumn N. Peters,[†] Persephone A. Jordano,[†] Jennifer A. Taylor,[†] Obadiah G. Reid,[*,‡,¶] and James R. Neilson[*,†,§]

†*Department of Chemistry, Colorado State University, Fort Collins, CO 80523, United States*

‡*Renewable and Sustainable Energy Institute, University of Colorado Boulder, Boulder, CO 80303, United States*

¶*Chemistry & Nanoscience Center, National Laboratory of the Rockies, Golden, CO 80401, United States*

§*School of Materials Science & Engineering, Colorado State University, Fort Collins, CO 80523, United States*

E-mail: obadiah.reid@NLR.gov; james.neilson@colostate.edu

Contents

Proton Nuclear Magnetic Resonance (NMR) Spectroscopy **S3**

Powder X-ray Diffraction (PXRD) Refinements **S12**

Dark Microwave Conductivity (DMC) **S15**

Time Resolved Microwave Conductivity (TRMC) Experiments **S31**

Proton Nuclear Magnetic Resonance (NMR) Spectroscopy

^{1}H NMR was used in determination of the ratio of ethylenediammonium dication (*en*) to the methylammonium (MA) cation. This process was followed according to Spanopolous et al. and their previous work with this system.[1] Quantification utilized the NMR signatures of the methyl (-CH_3) and ammonium (-NH_3^+) groups present in the MA cation and the methylene (-CH_2-) and ammonium (-NH_3^+) groups present in the *en* dication. The MA methyl proton peak appears at δ = 2.39 ppm and the ammonium protons at δ = 7.49 ppm, while in *en* the methylene proton peak appears at δ = 3.02 ppm and the ammonium protons at δ =7.77 ppm.

As expected based on the chemical structure of MA (3 protons in the methyl group and 3 protons in the ammonium group), a 1:1 integration ratio of the two peaks is observed. In *en* there is instead an observed integration ratio of 4:6 for methyl and ammonium groups, respectively. This ratio arises from the doubly protonated nature of *en* (4 methyl protons and 6 ammonium protons). To quantify the amount of *en* relative to MA, an equation that includes the two molecules was used while analyzing the ^{1}H NMR spectra: MA + *en* = 1. This equation maintains consistency with the standard perovskite formula, 113 (cation)BX_3 (cation = MA + *en*).[1] The total organic content was thus set equal to 1 (100%).[1] The integration values of either the two methyl/methylene proton peaks (from MA and *en*) or the two ammonium protons peaks can be set equal to 100%. Thus, by dividing the integration of the MA protons (either methyl or ammonium) by the total integration value of both species (total methyl/methylene or ammonium), the relative ratio of MA may be determined. Similarly, by dividing the *en* proton integration by the total integration value, the ratio of *en* was determined. This process was completed for all samples using the methyl/methylene proton ratio as these protons are less likely to experience hydrogen transfer effects in solution as opposed to their ammonium counterparts.

Using the outlined method, we were able to determine that we synthesized the follow-

ing members of the $MA_{1-x}en_xSn_{1-0.7x}I_{3-0.4x}$ (MA = methylammonium, x = ethylenediammonium) family: x = 0.00, 0.06, 0.10, 0.15, 0.25, 0.30, 0.34, 0.38.

The peak seen in the spectra at $\delta = 3.36$ ppm is attributed to H_2O from wet deuterated solvent. The triplet of peaks centered at $\delta = 7.09$ ppm were assigned to NH_4I based on previous reports.[2] We assume that a small amount of surface thermal degradation of the organic cations can occur during the annealing process leading to the appearance of these peaks.

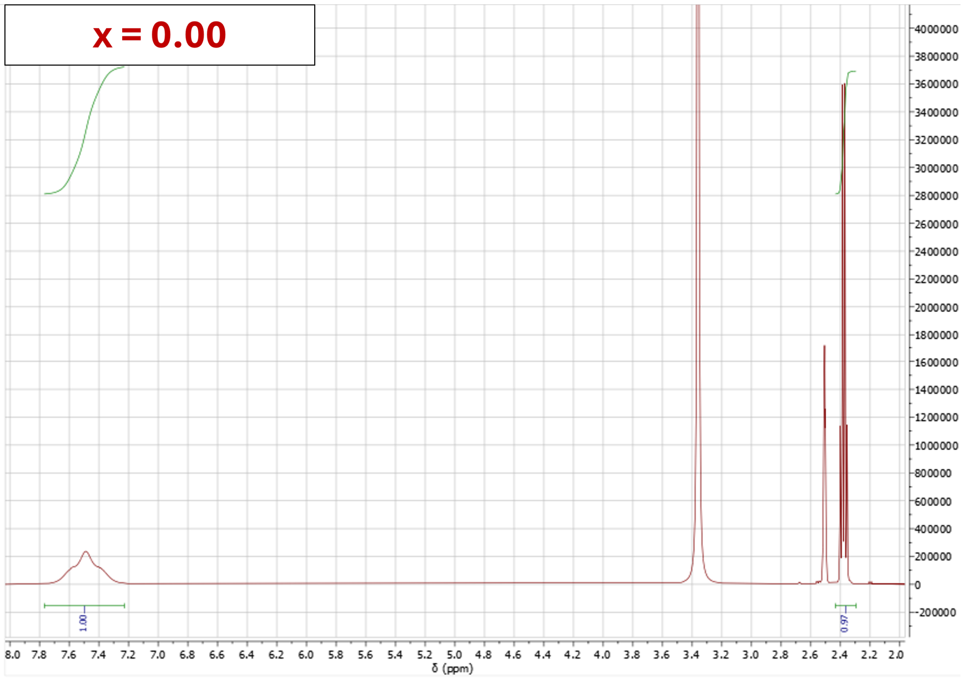

Figure S1: The proton (^{1}H NMR) spectra of $MA_{1-x}en_xSn_{1-0.7x}I_{3-0.4x}$ mechanochemical sample dissolved in DMSO-d_6. The relative amounts of *en* and MA cations was determined by comparing the intensities of the methylene signal of *en* ($\delta = 3.02$ ppm) methyl group signal of MA ($\delta = 2.39$ ppm) resulting in an $x = 0.00$ value.

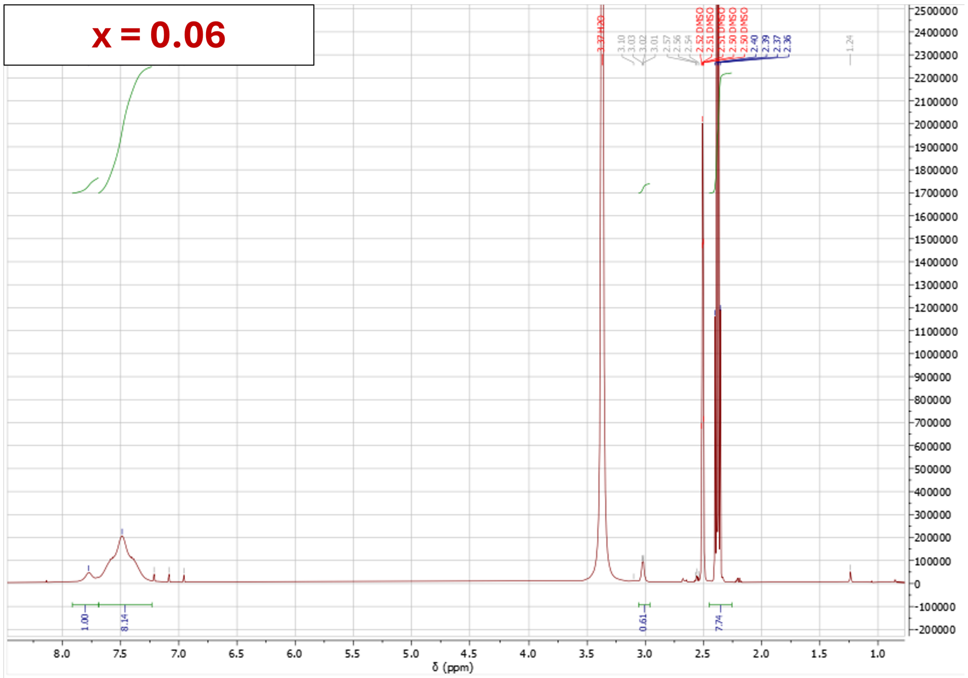

Figure S2: The proton (^{1}H NMR) spectra of $MA_{1-x}en_xSn_{1-0.7x}I_{3-0.4x}$ mechanochemical sample dissolved in DMSO-d_6. The relative amounts of *en* and MA cations was determined by comparing the intensities of the methylene signal of *en* (δ = 3.02 ppm) methyl group signal of MA (δ = 2.39 ppm) resulting in an x = 0.06 value.

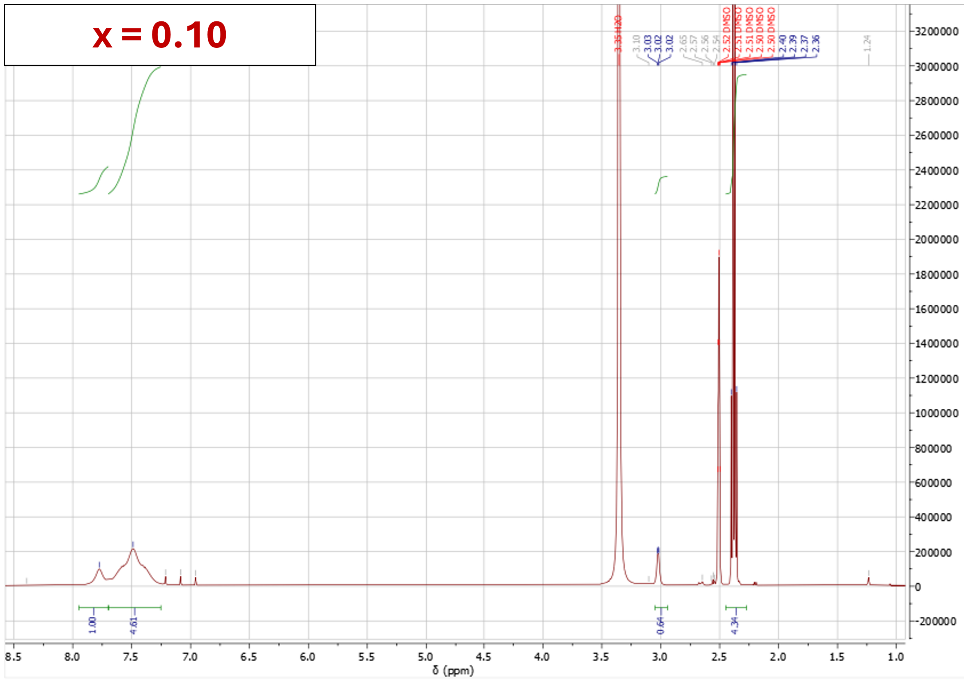

Figure S3: The proton (^{1}H NMR) spectra of $MA_{1-x}en_xSn_{1-0.7x}I_{3-0.4x}$ mechanochemical sample dissolved in DMSO-d_6. The relative amounts of *en* and MA cations was determined by comparing the intensities of the methylene signal of *en* (δ = 3.02 ppm) methyl group signal of MA (δ = 2.39 ppm) resulting in an x = 0.10 value.

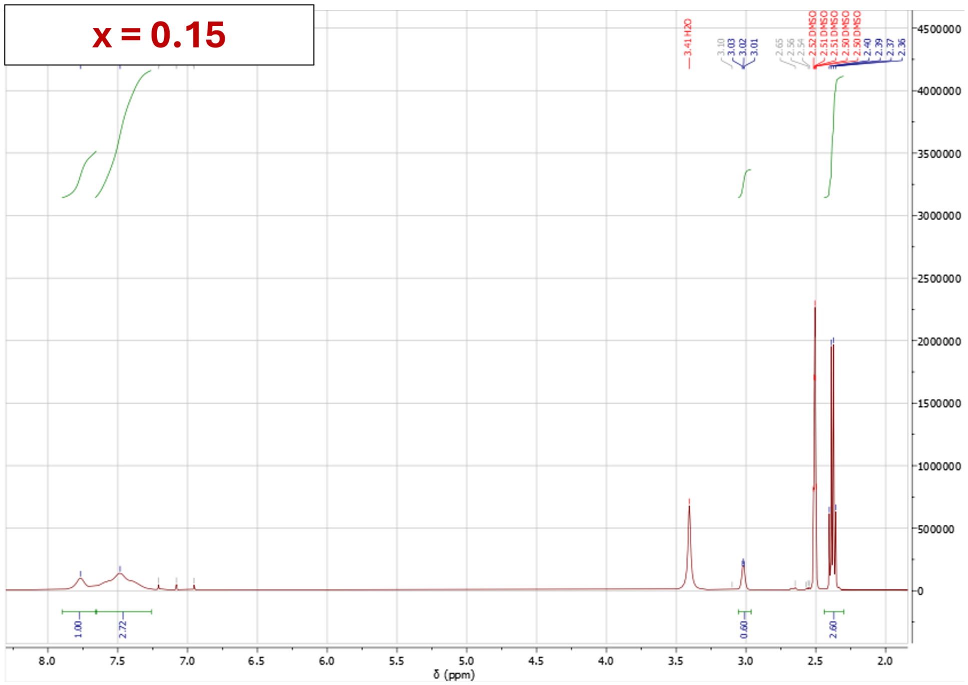

Figure S4: The proton (^{1}H NMR) spectra of $MA_{1-x}en_xSn_{1-0.7x}I_{3-0.4x}$ mechanochemical sample dissolved in DMSO-d_6. The relative amounts of *en* and MA cations was determined by comparing the intensities of the methylene signal of *en* (δ = 3.02 ppm) methyl group signal of MA (δ = 2.39 ppm) resulting in an x = 0.15 value.

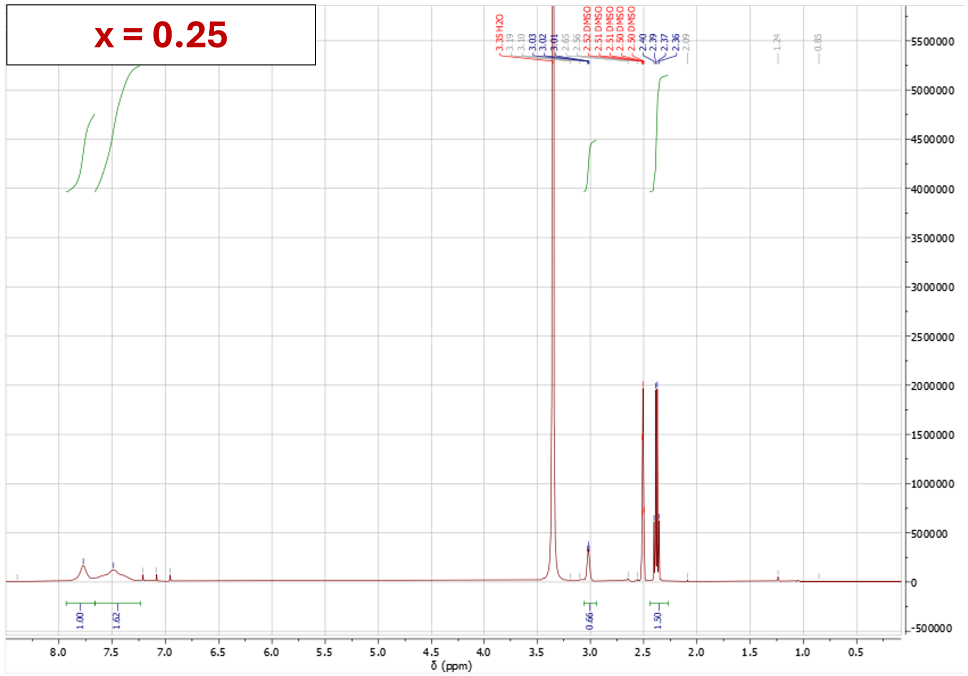

Figure S5: The proton (^{1}H NMR) spectra of $MA_{1-x}en_xSn_{1-0.7x}I_{3-0.4x}$ mechanochemical sample dissolved in DMSO-d_6. The relative amounts of *en* and MA cations was determined by comparing the intensities of the methylene signal of *en* (δ = 3.02 ppm) methyl group signal of MA (δ = 2.39 ppm) resulting in an x = 0.25 value.

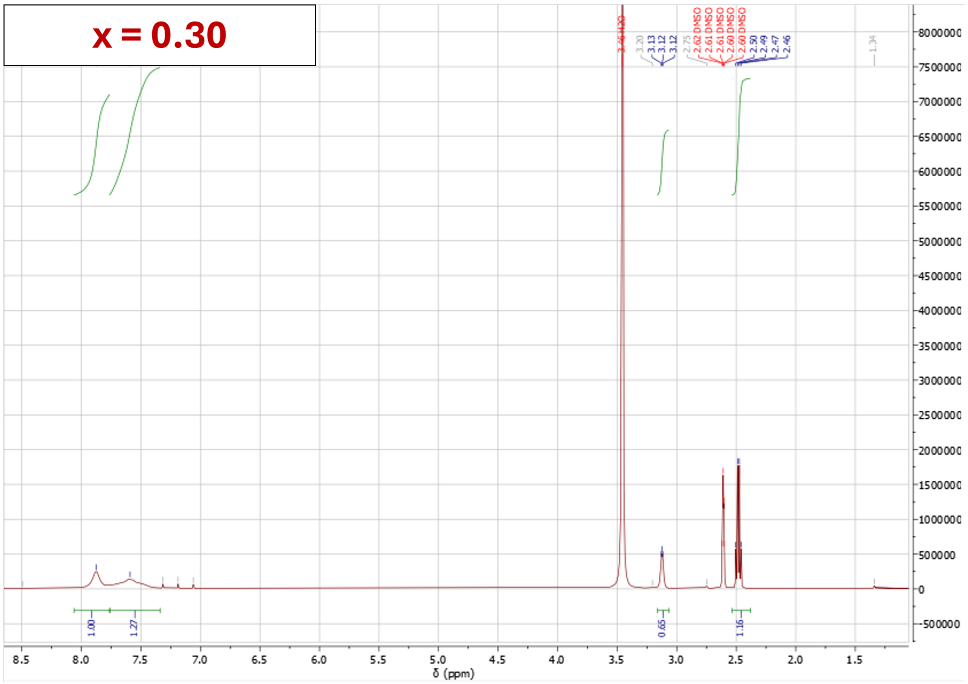

Figure S6: The proton (^{1}H NMR) spectra of $MA_{1-x}en_xSn_{1-0.7x}I_{3-0.4x}$ mechanochemical sample dissolved in DMSO-d_6. The relative amounts of *en* and MA cations was determined by comparing the intensities of the methylene signal of *en* (δ = 3.02 ppm) methyl group signal of MA (δ = 2.39 ppm) resulting in an x = 0.30 value.

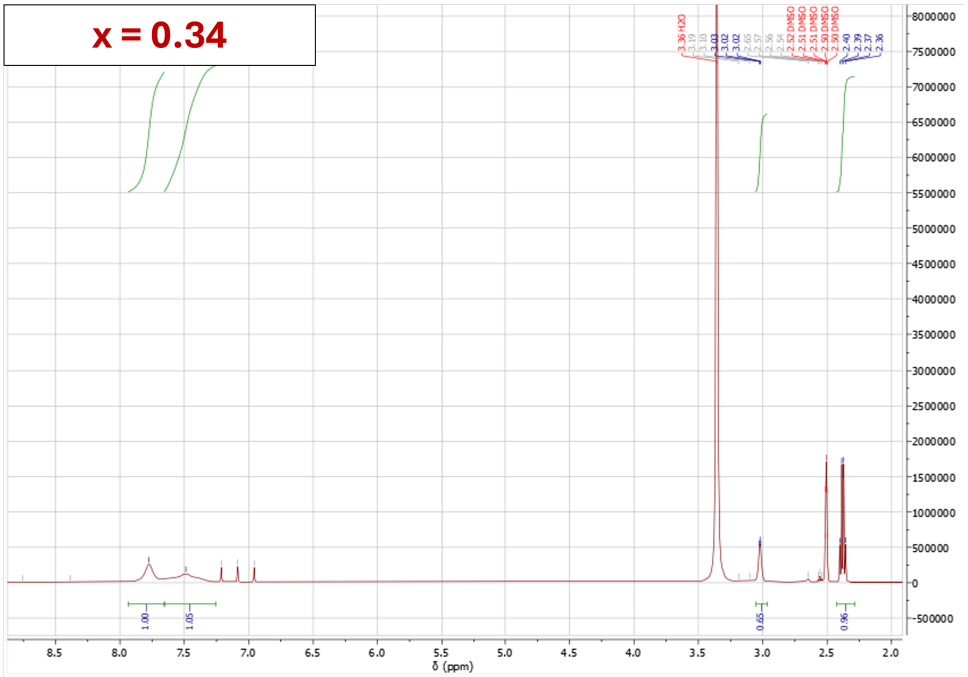

Figure S7: The proton (^{1}H NMR) spectra of $MA_{1-x}en_xSn_{1-0.7x}I_{3-0.4x}$ mechanochemical sample dissolved in DMSO-d_6. The relative amounts of *en* and MA cations was determined by comparing the intensities of the methylene signal of *en* (δ = 3.02 ppm) methyl group signal of MA (δ = 2.39 ppm) resulting in an x = 0.34 value.

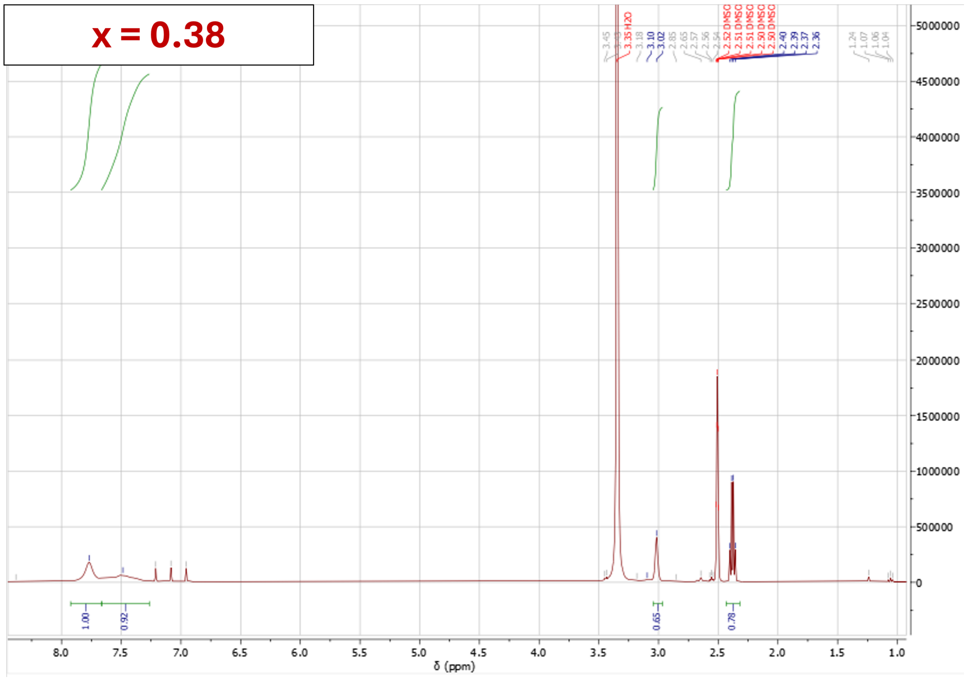

Figure S8: The proton (^{1}H NMR) spectra of $MA_{1-x}en_xSn_{1-0.7x}I_{3-0.4x}$ mechanochemical sample dissolved in DMSO-d_6. The relative amounts of *en* and MA cations was determined by comparing the intensities of the methylene signal of *en* (δ = 3.02 ppm) methyl group signal of MA (δ = 2.39 ppm) resulting in an x = 0.38 value.

Powder X-ray Diffraction (PXRD) Refinements

The experimental PXRD data was fit to the pristine α-$MASnI_3$ structure using a least-squares Rietveld refinement in TOPAS v6. We used a potassium ion (K^+) as a proxy for the organic cations in this structure as it is isoelectronic (18 electrons) to MA. The thermal parameters (B_{eq}) were refined helping to account for the differences in electron density between K^+ and the organics in the system. To account for instrument geometry (Bragg-Brentano) and sample preparation methods (powder samples on silicon wafers), both sample displacement and absorption (surface roughness) were refined. In these refinements, the background was fit as opposed to subtracted. The lattice parameters and microcrystalline size (Gaussian) were further refined in order to fit samples. The crystalline microstrain (Lorentzian) was an additional refinement factor that improved the fit of the broadened peaks (beyond instrumental broadening). From these fits, lattice parameters, and the corresponding unit cell volumes, were extracted. Silicon was used to spike samples, in a second diffraction collection experiment, as an internal standard for lattice parameter extraction. Lattice parameters reported correspond to the standard spiked refinements while peak fits and broadening parameters correspond to the fits from un-spiked samples to better capture the sample's peak shapes. Samples contained a very minimal amount of residual SnI_2 precursor, and the $x = 0.34$ and 0.38 samples contained a very small amount of NH_4I, which we believe to be the result of minimal surface degradation during the annealing step. Results from the PXRD Rietveld refinements can be seen in Tables S1 & S2.

Table S1: Structural and microstructural parameters obtained from Rietveld refinements of the synthetic development of $MA_{1-x}en_xSn_{1-0.7x}I_{3-0.4x}$ samples (Figure 2), with uncertainties in parentheses. Also included are the residual weight percents of the precursors SnI_2, enI_2, and MAI where applicable.

Sample	a (Å)	Crystallite size (Gaussian, nm)	Microcrystalline strain (Lorentzian)	wt% SnI_2	wt% enI_2	wt% MAI	R_{wp}	GOF
1-Step Mill	6.25014(4)	76.5(2.7)	0.762(24)	1.62	3.68	0.69	13.04	1.97
2-Step Mill	6.27475(6)	91.5(10)	1.01(43)	2.58	1.94	-	11.12	1.44
2-Step Mill + 100°C anneal	6.29884(7)	114(28)	0.384(27)	*	-	-	2.662	1.58
2-Step Mill + 150°C anneal	6.30537(5)	90.8(4.9)	0.127(19)	*	-	-	2.777	1.66
2-Step Mill + 200°C anneal	6.30599(4)	93.8(5.5)	0.074(20)	*	-	-	2.771	1.63

* We note that the annealed samples were measured using kapton tape to ensure the samples were measured air-free in these initial trials. Because of this, the higher background made quantifying the expected small residual of SnI_2 impossible. With no visible peak shouldering where residual enI_2 and MAI reflections appear, we are confident that these samples do not contain unreacted organic precursor.

Table S2: Structural and microstructural parameters obtained from Rietveld refinements of $MA_{1-x}en_xSn_{1-0.7x}I_{3-0.4x}$ (MA = methylammonium, x = ethylenediammonium) where x = 0.00, 0.06, 0.10, 0.15, 0.25, 0.30, 0.34, and 0.38 (Figures 3 & S9) in TOPAS v6 with uncertainties in parentheses. Also included are the residual weight percents of SnI_2 and the NH_4I that appeared in higher x samples.

x $MA_{(1-x)}en_xSn_{(1-0.7x)}I_{(3-0.4x)}$	a (Å)	Crystallite size (Gaussian, nm)	Microcrystalline strain (Lorentzian)	wt% SnI_2	wt% NH_4I	R_{wp}	GOF
0.00	6.245997(8)	136(3)	0.1385(7)	1.10	-	14.6	2.02
0.06	6.255246(6)	138(2)	0.0359(16)	1.51	-	14.9	2.21
0.10	6.268060(6)	159(3)	0.1065(7)	2.00	-	15.11	2.46
0.15	6.278824(6)	164(4)	0.0941(8)	2.00	-	14.9	2.43
0.25	6.304093(8)	154(4)	0.1341(8)	2.06	-	14.0	2.14
0.30	6.318624(7)	123(2)	0.0387(24)	2.66	-	13.5	2.10
0.34	6.333127(8)	139(3)	0.0568(16)	2.31	1.84	12.7	1.94
0.38	6.343959(9)	150(4)	0.1577(89)	2.56	1.79	12.9	2.03

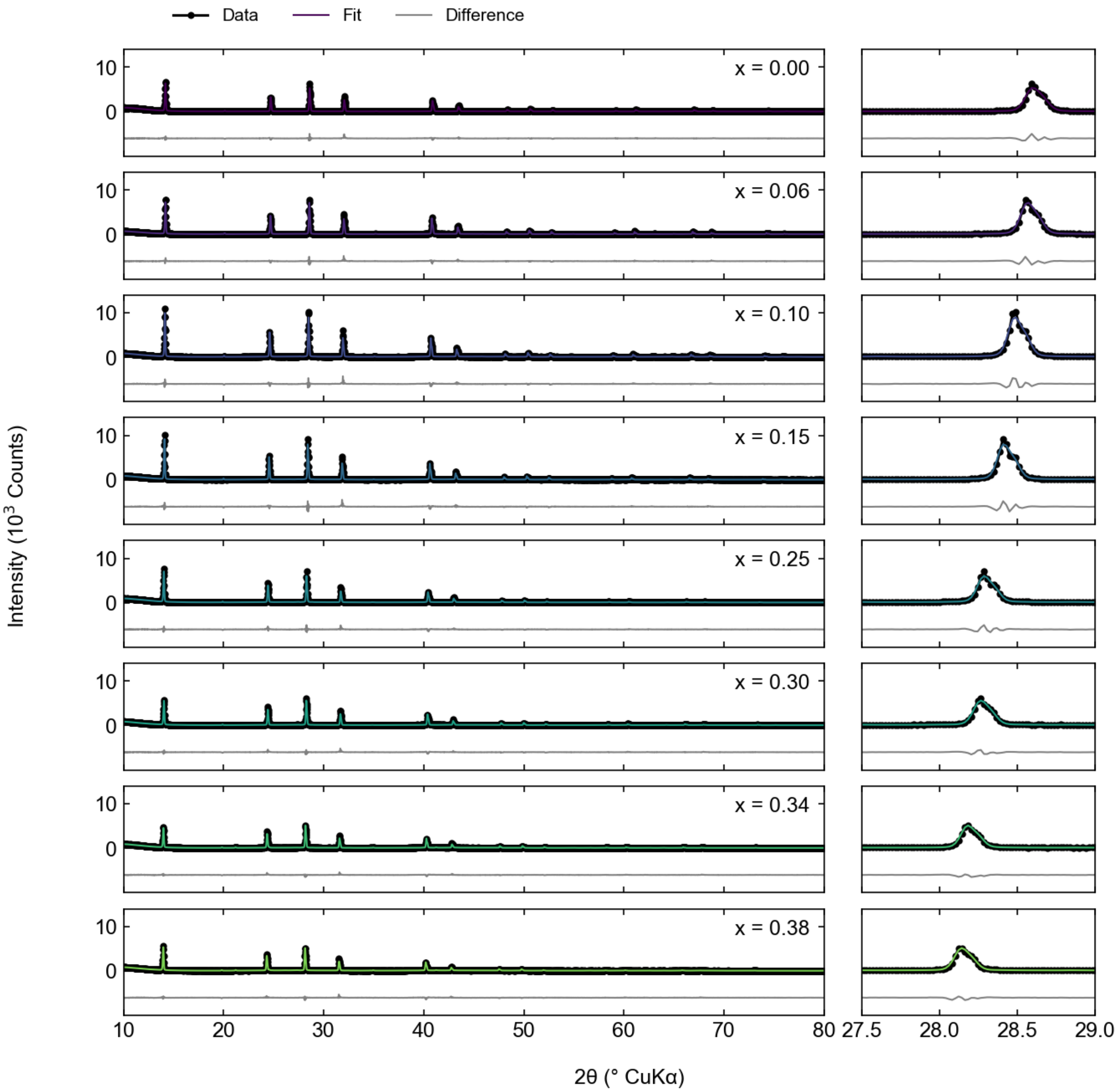

Figure S9: The PXRD data of $MA_{1-x}en_xSn_{1-0.7x}I_{3-0.4x}$ samples where $x = 0.00$, 0.06, 0.10, 0.15, 0.25, 0.30, 0.34, and 0.38. The raw data is shown in black, the fit in gradient colors, and the difference curve between the two after a least-squares, Rietveld refinement is in gray. Zoomed-in portions of these plots in the right column show the systematic peak shift (unit cell volume increase) that occurs as more x is incorporated.

Dark Microwave Conductivity (DMC)

A block diagram of the new instrument is shown in Figure S10. This design largely conforms to prior circuits,[3,4] but uses more modern components in several areas. Of particular note are the use of a LabJack U6 Pro both to provide a tuning voltage and read the detector voltage, and the use of the Pasternack (PE80T6021) tunnel diode detector.

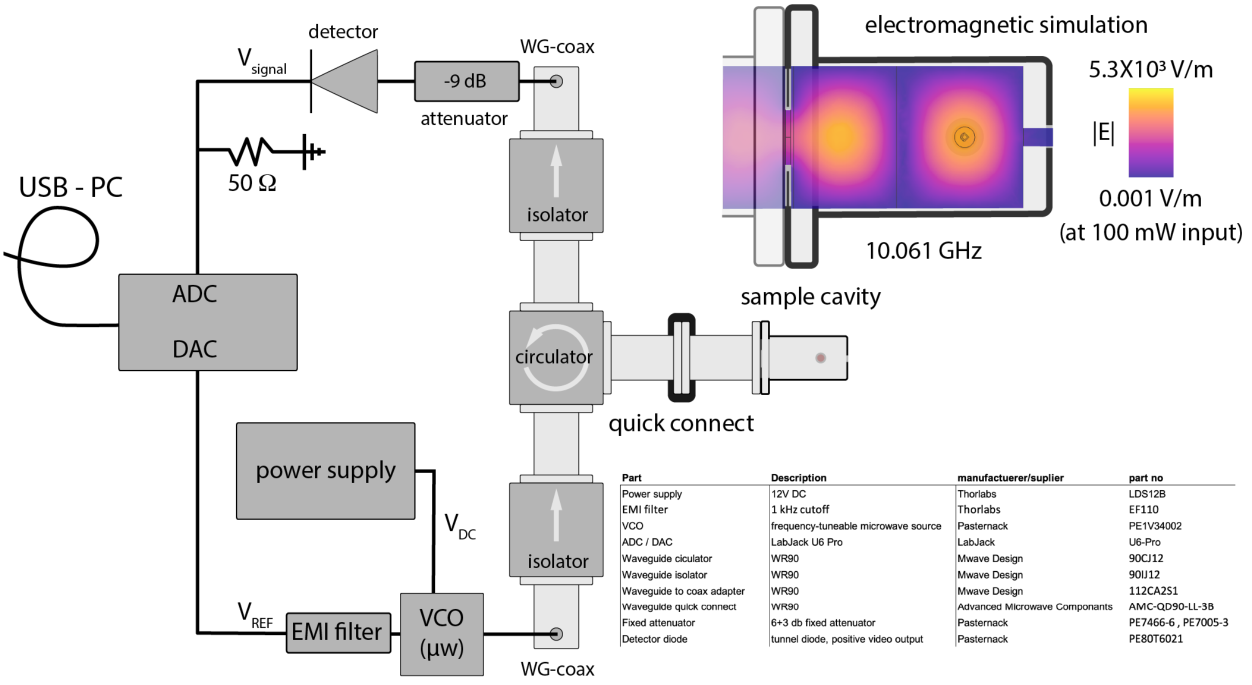

Part	Description	manufactuerer/suplier	part no
Power supply	12V DC	Thorlabs	LDS12B
EMI filter	1 kHz cutoff	Thorlabs	EF110
VCO	frequency-tuneable microwave source	Pasternack	PE1V34002
ADC / DAC	LabJack U6 Pro	LabJack	U6-Pro
Waveguide ciculator	WR90	Mwave Design	90CJ12
Waveguide isolator	WR90	Mwave Design	90IJ12
Waveguide to coax adapter	WR90	Mwave Design	112CA2S1
Waveguide quick connect	WR90	Advanced Microwave Componants	AMC-QD90-LL-3B
Fixed attenuator	6+3 db fixed attenuator	Pasternack	PE7466-6 , PE7005-3
Detector diode	tunnel diode, positive video output	Pasternack	PE80T6021

Figure S10: A block diagram of the new dark microwave conductivity system constructed at CSU, including a list of the most important active components and a simulation of the microwave electric field amplitude in the cavity. Note that the sample is contained in a borosilicate capillary tube and positioned at one of the two electric field maxima of the TE_{102} mode.

The LabJack provides a much more compact and economical means of controlling the voltage-controlled oscillator (VCO, Pasternack PE1V34002, 21 dBm) and reading the microwave detector voltage than instruments we have used previously, while the chosen detector provides comparable sensitivity to the traditional waveguide-integrated Schottky diode detectors – a hard thing to find in coaxial components. The attenuators employed between the detector and the microwave circuit are needed to keep this detector from

going into saturation.

Dark conductivity measurements were analyzed using resonance curves (microwave power reflectance vs. frequency) obtained from a brass terminating plate at the position of the cavity iris, an empty capillary sample tube in the cavity, and sample packed into the capillary tube, using modified Teflon ferrules to center the tubes in the cavity in each case. The resonance curves were fit with a finite-element electromagnetic model[3,5,6] to extract equilibrium complex dielectric constant, which can also be expressed as a conductivity using the relationship: $\sigma' = \varepsilon_r''(\omega\varepsilon_0)$, $\sigma'' = \varepsilon_r'(\omega\varepsilon_0)$, where $'$ indicates the real part, and $''$ the imaginary part. The measured values were corrected to account for sample filling fraction, f, of the tubes using the simplest possible effective medium approximation: that the measured properties of the powder arise from a volume weighted average of air ($\epsilon_r = 1 - 0i$) and the semiconductor. Representative samples (x = 0.00, 0.25) were packed in 5 capillaries each and an average filling fraction, f, was determined to be ~40% and was calculated using the measured powder density (ρ_{powder}) and the experimental crystal density ($\rho_{crystal}$): $f = \rho_{powder}/\rho_{crystal}$. The real part of the sample permittivity is then calculated as: $\varepsilon_r' = \frac{\varepsilon'_{powder}(1-f)}{f}$ and the imaginary part as: $\varepsilon_r'' = \frac{\varepsilon''_{powder}}{f}$.

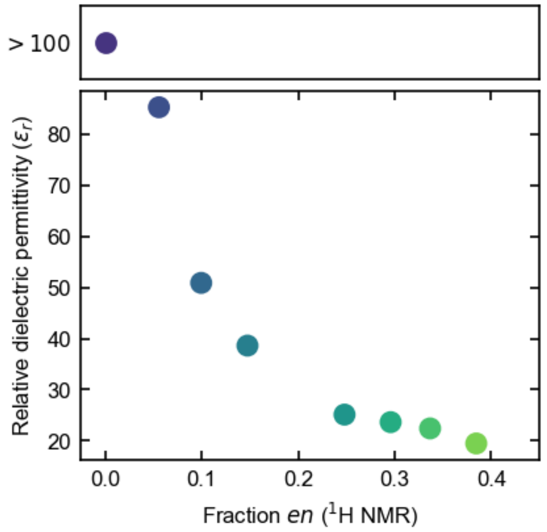

Figure S11: Extrapolated dielectric permittivity from dark microwave conductivity measurements for $MA_{1-x}en_xSn_{1-0.7x}I_{3-0.4x}$ $0.00 \leq x \leq 0.38$.

Propagation of Uncertainty for Packing Fraction–Corrected Conductivity and Carrier Density

Packing Fraction Determination

The packing fraction, f, accounts for incomplete filling of the capillary volume by the powder sample. For each independently packed capillary, i, the sample packing density, $d_{\text{packing},i}$, was calculated as

$$d_{\text{packing},i} = \frac{m_i}{V_i}, \tag{S1}$$

where m_i is the sample mass obtained from the difference between filled and empty capillary weights, and V_i is the sample volume calculated assuming a cylindrical geometry using the measured sample height and the capillary inner diameter. The packing fraction for each capillary was then calculated as,

$$f_i = \frac{d_{\text{packing},i}}{d_{\text{exp}}}, \tag{S2}$$

where d_{exp} is the experimentally measured bulk density of the material. Ten independently packed capillaries were used to determine the packing fraction. The mean packing fraction,

$$\bar{f} = \frac{1}{N}\sum_{i=1}^{N} f_i, \tag{S3}$$

was used to correct all permittivity and conductivity values. Because this value was applied to measurements obtained from a separate dataset, the uncertainty associated with the packing fraction was taken as the sample standard deviation of the ten measurements rather than the standard deviation of the mean,

$$u_f = s_f. \tag{S4}$$

Packing Fraction Corrections

The imaginary permittivity and real conductivity were corrected for using the average packing fraction, f, according to,

$$X_{\mathrm{corr}} = \frac{X_{\mathrm{meas}}}{f}, \tag{S5}$$

where X represents either ε'' or σ. The real permittivity was corrected for incomplete packing according to,

$$\varepsilon'_{corr} = \frac{\varepsilon'_m - (1-f)}{f}, \tag{S6}$$

where ε'_m is the measured real permittivity, f is the mean packing fraction, and ε'_{corr} is the corrected real permittivity.

The largest source of uncertainty in determination of the unscaled (not corrected) permittivities (real $u_{\varepsilon'_{\mathrm{m}}}$ and imaginary $u_{\varepsilon''_{\mathrm{m}}}$) is likely the uncertainty from fitting the resonance curves from an interpolated lookup table from electromagnetic simulations.

The uncertainty in the corrected real permittivity, $u_{\varepsilon'_{\mathrm{corr}}}$, was calculated using standard propagation of uncertainty for uncorrelated variables and can be written in the standard form,

$$u_{\varepsilon'_{\mathrm{corr}}} = \frac{1}{|f|}\sqrt{u^2_{\varepsilon'_m} + \left(\frac{\varepsilon'_m - 1}{f}\right)^2 u_f^2}. \tag{S7}$$

The imaginary permittivity was corrected for incomplete packing according to,

$$\varepsilon''_{corr} = \frac{\varepsilon''_m}{f}, \tag{S8}$$

where ε''_m is the measured imaginary permittivity, f is the mean packing fraction, and ε''_{corr}

is the corrected imaginary permittivity.

The uncertainty in the corrected imaginary permittivity, $u_{\varepsilon''_{\text{corr}}}$, was calculated using the standard propagation of uncertainty for a quotient of uncorrelated variables, expressed in relative form,

$$u_{\varepsilon''_{\text{corr}}} = |\varepsilon''_{\text{corr}}| \sqrt{\left(\frac{u_{\varepsilon''_m}}{\varepsilon''_m}\right)^2 + \left(\frac{u_f}{f}\right)^2}. \tag{S9}$$

The real component of the raw dark microwave conductivity, σ_m, was calculated from the measured imaginary permittivity according to,

$$\sigma_m = \varepsilon_0 \omega \varepsilon''_m, \tag{S10}$$

where ε_0 is the vacuum permittivity and ω is the angular frequency determined by the instrument parameters. Both ε_0 and ω were treated as exact constants. Under this assumption, the uncertainty in the uncorrected real conductivity, u_{σ_m}, propagates directly from the uncertainty in the measured imaginary permittivity,

$$\frac{u_{\sigma_m}}{\sigma_m} = \frac{u_{\varepsilon''_m}}{\varepsilon''_m}. \tag{S11}$$

The uncertainty in the corrected real conductivity, $u_{\sigma_{\text{corr}}}$, was calculated using the standard propagation of uncertainty for a quotient of uncorrelated variables, written in relative form,

$$u_{\sigma_{\text{corr}}} = |\sigma_{\text{corr}}| \sqrt{\left(\frac{u_{\sigma_m}}{\sigma_m}\right)^2 + \left(\frac{u_f}{f}\right)^2}. \tag{S12}$$

These expressions follow directly from the general law of propagation of uncertainty and accounts for contributions from both the measured permittivity and the uncertainty in the packing fraction. All quantities were assumed to be uncorrelated.

Carrier Density Calculation & Carrier Lifetime Uncertainty

The charge carrier density, n, was estimated from the packing-fraction-corrected dark conductivity using,

$$n = \frac{\sigma_{\mathrm{corr}}}{e\mu}, \tag{S13}$$

where σ_{corr} is the corrected real conductivity, e is the elementary charge, and μ is the charge carrier mobility obtained from time-resolved microwave conductivity measurements. The elementary charge was treated as an exact constant.

Because both σ_{corr} and μ have associated uncertainties and were assumed to be uncorrelated, the uncertainty in the carrier density, u_n, was calculated using the standard propagation of uncertainty for a quotient of independent variables. Expressed in relative form,

$$\frac{u_n}{n} = \sqrt{\left(\frac{u_{\sigma_{\mathrm{corr}}}}{\sigma_{\mathrm{corr}}}\right)^2 + \left(\frac{u_\mu}{\mu}\right)^2}. \tag{S14}$$

Equivalently, the absolute uncertainty in the carrier density can be written as,

$$u_n = |n| \sqrt{\left(\frac{u_{\sigma_{\mathrm{corr}}}}{\sigma_{\mathrm{corr}}}\right)^2 + \left(\frac{u_\mu}{\mu}\right)^2}. \tag{S15}$$

The uncertainty in μ (u_μ) was calculated from the covariance matrix (C) generated during fitting of the multi-exponential decay function to the data, using the square-root of the diagonal elements to calculate the uncertainty of each output parameter: $u_{P_i} = \sqrt{C[i,i]}$. Weights were provided to the fit procedure, which are the standard deviation of the mean at every time point in a microwave conductivity transient derived from averaging the experimental data. These uncertainties thus reflect random noise on the microwave probe signal as well as the laser pulse intensity. The yield-mobility product ($\phi\Sigma\mu(t = 0)$) is obtained by summing over each exponential prefactors (A_i) in the fit

according to:

$$\phi \Sigma \mu(t=0) = A_{tot} = \Sigma_i A_i. \tag{S16}$$

We assume in this work that $\phi = 1$, thus the overall uncertainty in the mobility is found by pooling the uncertainty in each A_i according to:

$$u_\mu = \sqrt{\Sigma_i u_{A_i}^2}. \tag{S17}$$

Uncertainties in the average lifetime ($\langle\tau\rangle$, $u_{\langle\tau\rangle}$) are derived from the same set of fit parameters, and obtained through a similar procedure, but weighting the uncertainty contributions by their relative contribution to the total amplitude, $A_{tot} = \phi\Sigma\mu(t = 0)$ using:

$$u_{\langle\tau\rangle} = \sqrt{\sum_i [u_{\tau_i}^2 \frac{A_i}{A_{tot}}]}, \tag{S18}$$

since the average lifetime is calculated as:

$$\langle\tau\rangle = \sum_i [\tau_i \frac{A_i}{A_{tot}}]. \tag{S19}$$

The resulting carrier densities and their propagated uncertainties are reported in Tables S3 and S4.

Table S3: Raw dielectric properties and measured conductivity prior to f-correction, with uncertainties.

Averaged packing fraction = 0.4067 ± 0.0304 (relative standard uncertainty = 0.0747)			
**x** $MA_{(1-x)}en_xSn_{(1-0.7x)}I_{(3-0.4x)}$	**Raw** ε''_m **± unc.**	**Raw** ε'_m **± unc.**	**Raw** σ_m **($S\,m^{-1}$) ± unc.**
0.00	–	–	–
0.06	22.22 ± 0.12	35.31 ± 0.15	12.06 ± 0.068
0.10	5.53 ± 0.02	21.33 ± 0.02	3.05 ± 0.0095
0.15	3.52 ± 0.01	15.57 ± 0.01	1.95 ± 0.0039
0.25	0.658 ± 0.001	10.77 ± 0.005	0.367 ± 0.00073
0.30	0.485 ± 0.001	10.19 ± 0.006	0.271 ± 0.00070
0.34	0.460 ± 0.001	9.68 ± 0.006	0.257 ± 0.00073
0.38	0.443 ± 0.001	8.49 ± 0.006	0.248 ± 0.00074

Table S4: f-corrected dielectric properties, conductivity, and calculated carrier density with propagated uncertainties.

**x** $MA_{(1-x)}en_xSn_{(1-0.7x)}I_{(3-0.4x)}$	ε'_{corr} **(real) ± unc.**	ε''_{corr} **(imag.) ± unc.**	σ_{corr} **($S\,m^{-1}$) ± unc.**
0.00	–	–	–
0.06	85.36 ± 6.31	54.64 ± 4.10	29.64 ± 2.22
0.10	50.99 ± 3.74	13.60 ± 1.02	7.493 ± 0.560
0.15	36.84 ± 2.68	8.66 ± 0.647	4.803 ± 0.359
0.25	25.03 ± 1.80	1.62 ± 0.121	0.903 ± 0.068
0.30	23.59 ± 1.69	1.19 ± 0.089	0.665 ± 0.050
0.34	22.33 ± 1.59	1.13 ± 0.085	0.633 ± 0.047
0.38	19.41 ± 1.38	1.09 ± 0.081	0.609 ± 0.046

Table S5: Carrier transport properties (mobility, lifetime, and calculated carrier density) with associated or propagated uncertainty.

**x** $\mathbf{MA_{(1-x)}en_xSn_{(1-0.7x)}I_{(3-0.4x)}}$	**Mobility** $\mathbf{(m^2V^{-1}s^{-1}) \pm}$ **unc.**	**Lifetime (ns)** $\pm$ **unc.**	**Carrier density** $\mathbf{(cm^{-3}) \pm}$ **unc.**
0.06	1.341 $\pm$ 0.0163	4.068 $\pm$ 0.0932	$1.39\times10^{18} \pm 1.05\times10^{17}$
0.10	0.892 $\pm$ 0.0184	3.864 $\pm$ 0.5594	$5.33\times10^{17} \pm 4.14\times10^{16}$
0.15	0.572 $\pm$ 0.228	13.21 $\pm$ 24.00	$5.36\times10^{17} \pm 2.17\times10^{17}$
0.25	0.0706 $\pm$ 0.000263	271.17 $\pm$ 3.63	$8.69\times10^{17} \pm 6.51\times10^{16}$
0.30	0.0266 $\pm$ 0.000109	232.47 $\pm$ 5.90	$1.73\times10^{18} \pm 1.30\times10^{17}$
0.34	0.00310 $\pm$ 0.0000274	39.55 $\pm$ 2.00	$1.48\times10^{19} \pm 1.12\times10^{18}$
0.38	0.00701 $\pm$ 0.000112	84.32 $\pm$ 4.56	$4.38\times10^{18} \pm 3.35\times10^{17}$

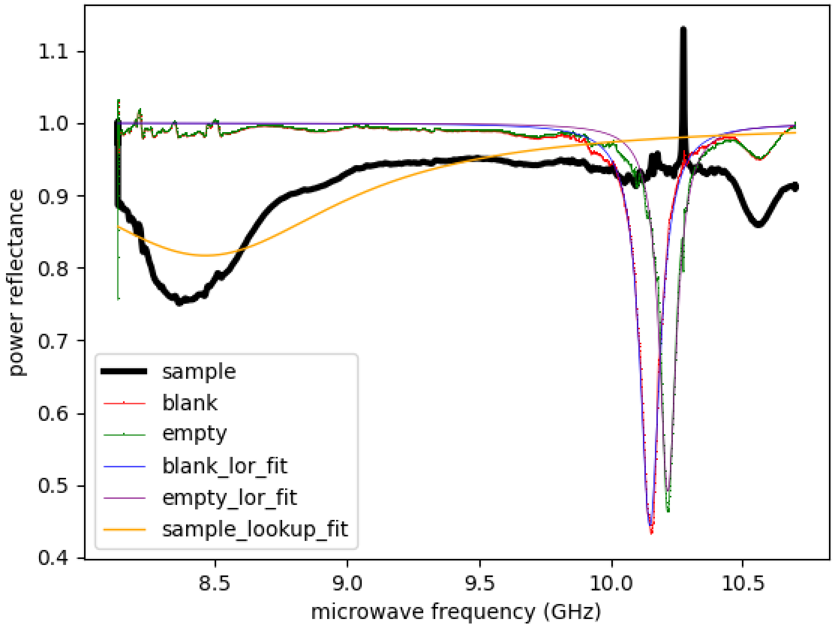

Figure S12: Resonance curves for $MA_{1-x}en_xSn_{1-0.7x}I_{3-0.4x}$ $x = 0.00$ and the empty tube (borosilicate capillary), where the x-axis represents the frequency (GHz) and the y-axis shows the power reflection coefficient. The reflectance data for the empty cavity (empty, green line), the cavity with an empty tube (blank, red line), and the sample (sample, black line) are shown. Each curve is fit with a Lorentzian, illustrated as blank_lor_fit (blue line), empty_lor_fit (purple line) and sample_lookup_fit (orange line). The latter is constrained by an electromagnetic simulation of the cavity response, providing values for the material's equilibrium conductivity and the relative dielectric constant. **Note:** the poor fit to the data in this instance is because this sample lies outside the measurable dynamic range for this instrument and/or sample configuration.

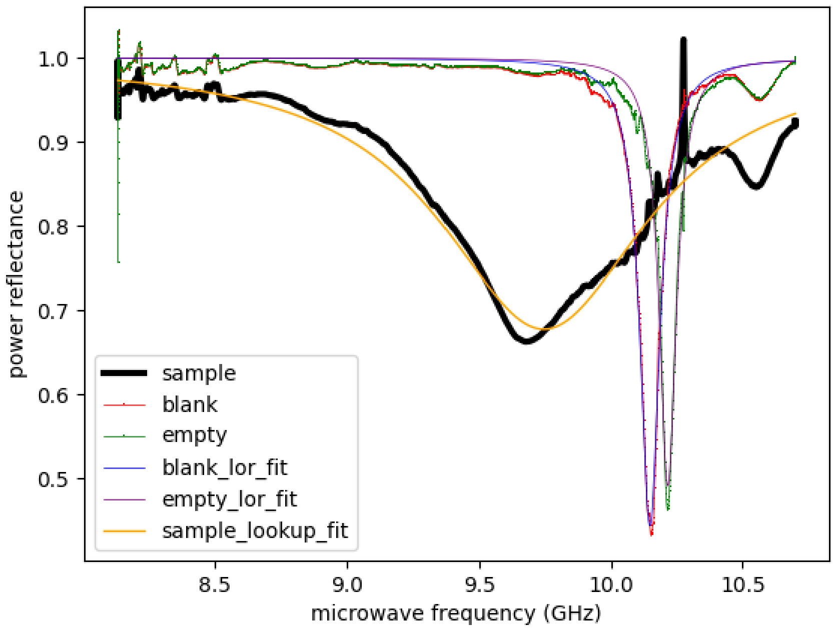

Figure S13: Resonance curves for $MA_{1-x}en_xSn_{1-0.7x}I_{3-0.4x}$ $x = 0.06$ and the empty tube (borosilicate capillary), where the x-axis represents the frequency (GHz) and the y-axis shows the power reflection coefficient. The reflectance data for the empty cavity (empty, green line), the cavity with an empty tube (blank, red line), and the sample (sample, black line) are shown. Each curve is fit with a Lorentzian, illustrated as blank_lor_fit (blue line), empty_lor_fit (purple line) and sample_lookup_fit (orange line). The latter is constrained by an electromagnetic simulation of the cavity response, providing values for the material's equilibrium conductivity and the relative dielectric constant.

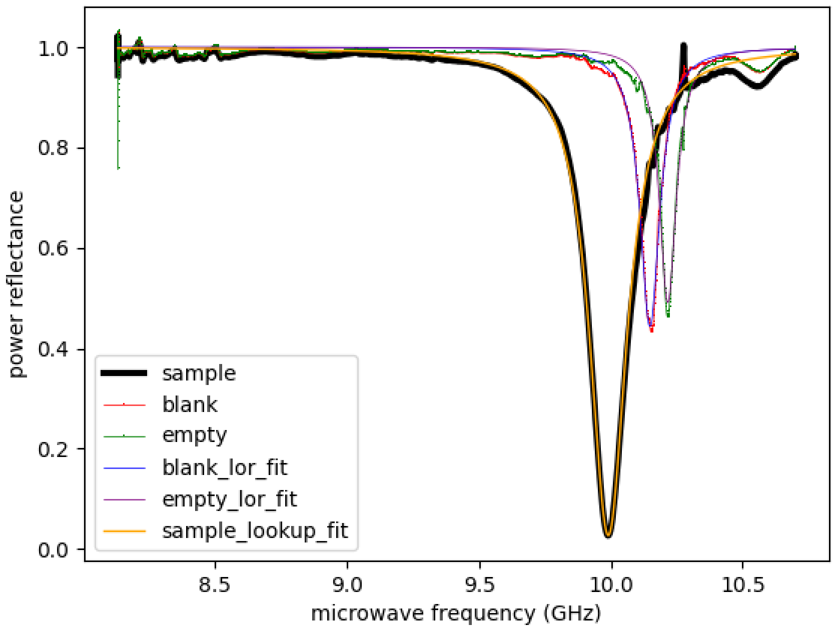

Figure S14: Resonance curves for $MA_{1-x}en_xSn_{1-0.7x}I_{3-0.4x}$ $x = 0.10$ and the empty tube (borosilicate capillary), where the x-axis represents the frequency (GHz) and the y-axis shows the power reflection coefficient. The reflectance data for the empty cavity (empty, green line), the cavity with an empty tube (blank, red line), and the sample (sample, black line) are shown. Each curve is fit with a Lorentzian, illustrated as blank_lor_fit (blue line), empty_lor_fit (purple line) and sample_lookup_fit (orange line). The latter is constrained by an electromagnetic simulation of the cavity response, providing values for the material's equilibrium conductivity and the relative dielectric constant.

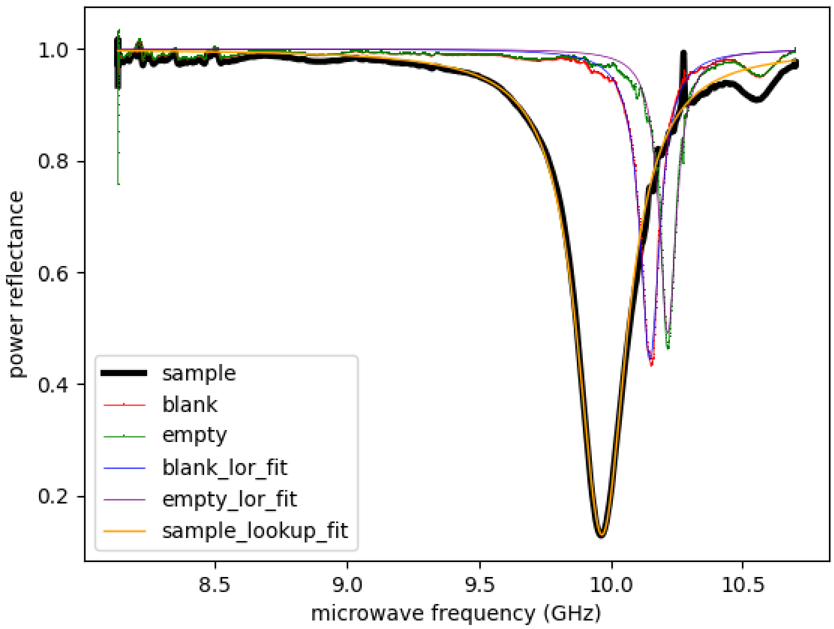

Figure S15: Resonance curves for $MA_{1-x}en_xSn_{1-0.7x}I_{3-0.4x}$ $x = 0.15$ and the empty tube (borosilicate capillary), where the x-axis represents the frequency (GHz) and the y-axis shows the power reflection coefficient. The reflectance data for the empty cavity (empty, green line), the cavity with an empty tube (blank, red line), and the sample (sample, black line) are shown. Each curve is fit with a Lorentzian, illustrated as blank_lor_fit (blue line), empty_lor_fit (purple line) and sample_lookup_fit (orange line). The latter is constrained by an electromagnetic simulation of the cavity response, providing values for the material's equilibrium conductivity and the relative dielectric constant.

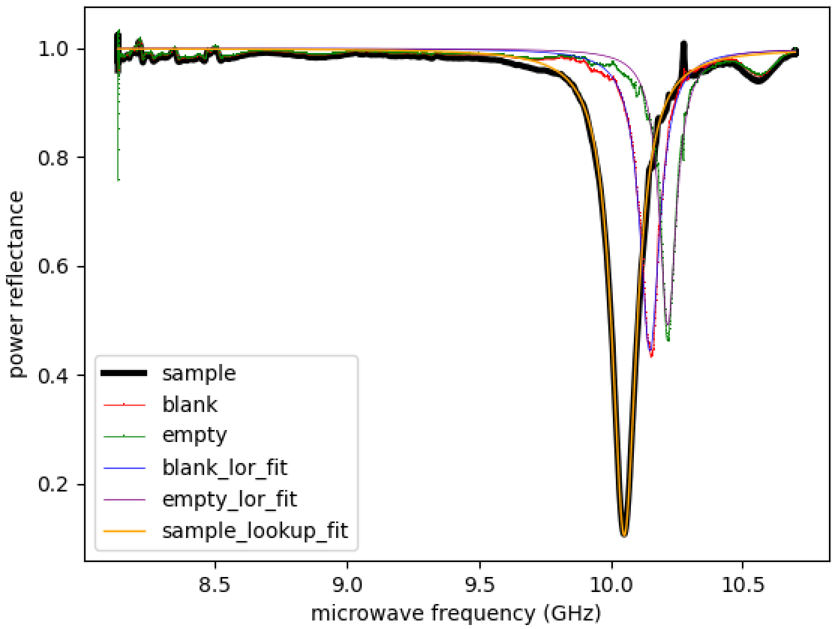

Figure S16: Resonance curves for $MA_{1-x}en_xSn_{1-0.7x}I_{3-0.4x}$ $x = 0.25$ and the empty tube (borosilicate capillary), where the x-axis represents the frequency (GHz) and the y-axis shows the power reflection coefficient. The reflectance data for the empty cavity (empty, green line), the cavity with an empty tube (blank, red line), and the sample (sample, black line) are shown. Each curve is fit with a Lorentzian, illustrated as blank_lor_fit (blue line), empty_lor_fit (purple line) and sample_lookup_fit (orange line). The latter is constrained by an electromagnetic simulation of the cavity response, providing values for the material's equilibrium conductivity and the relative dielectric constant.

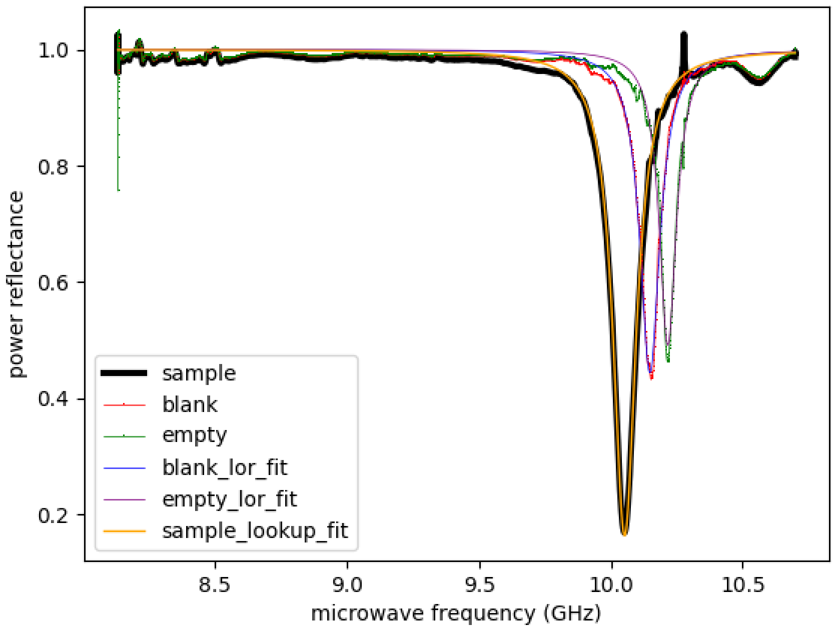

Figure S17: Resonance curves for $MA_{1-x}en_xSn_{1-0.7x}I_{3-0.4x}$ $x = 0.30$ and the empty tube (borosilicate capillary), where the x-axis represents the frequency (GHz) and the y-axis shows the power reflection coefficient. The reflectance data for the empty cavity (empty, green line), the cavity with an empty tube (blank, red line), and the sample (sample, black line) are shown. Each curve is fit with a Lorentzian, illustrated as blank_lor_fit (blue line), empty_lor_fit (purple line) and sample_lookup_fit (orange line). The latter is constrained by an electromagnetic simulation of the cavity response, providing values for the material's equilibrium conductivity and the relative dielectric constant.

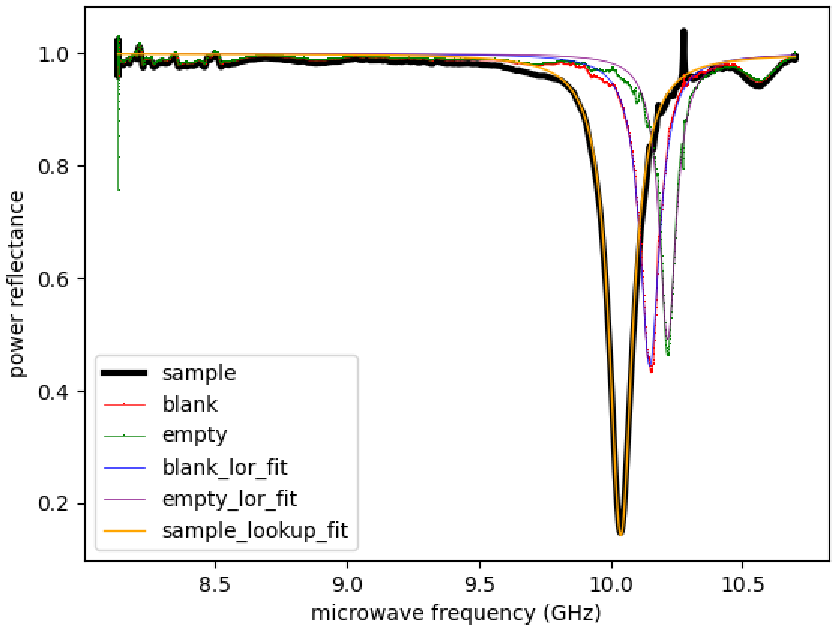

Figure S18: Resonance curves for $MA_{1-x}en_xSn_{1-0.7x}I_{3-0.4x}$ $x = 0.34$ and the empty tube (borosilicate capillary), where the x-axis represents the frequency (GHz) and the y-axis shows the power reflection coefficient. The reflectance data for the empty cavity (empty, green line), the cavity with an empty tube (blank, red line), and the sample (sample, black line) are shown. Each curve is fit with a Lorentzian, illustrated as blank_lor_fit (blue line), empty_lor_fit (purple line) and sample_lookup_fit (orange line). The latter is constrained by an electromagnetic simulation of the cavity response, providing values for the material's equilibrium conductivity and the relative dielectric constant.

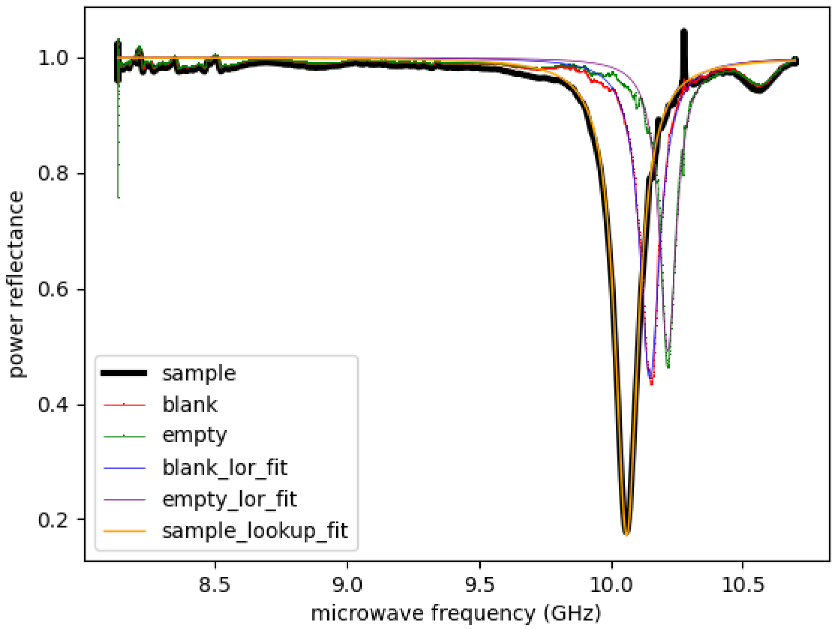

Figure S19: Resonance curves for $MA_{1-x}en_xSn_{1-0.7x}I_{3-0.4x}$ x = 0.38 and the empty tube (borosilicate capillary), where the x-axis represents the frequency (GHz) and the y-axis shows the power reflection coefficient. The reflectance data for the empty cavity (empty, green line), the cavity with an empty tube (blank, red line), and the sample (sample, black line) are shown. Each curve is fit with a Lorentzian, illustrated as blank_lor_fit (blue line), empty_lor_fit (purple line) and sample_lookup_fit (orange line). The latter is constrained by an electromagnetic simulation of the cavity response, providing values for the material's equilibrium conductivity and the relative dielectric constant.

Time Resolved Microwave Conductivity (TRMC) Experiments

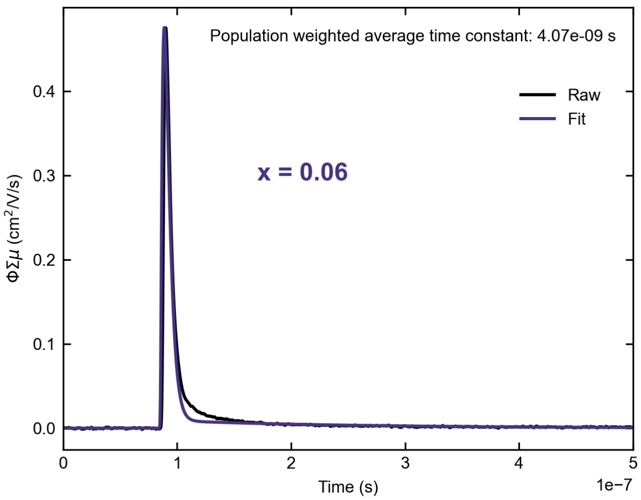

Figure S20: The plot shows raw (black line) and fitted (colored line) yield mobility product ($\phi\Sigma\mu$) for $MA_{1-x}en_xSn_{1-0.7x}I_{3-0.4x}$ $x = 0.06$.

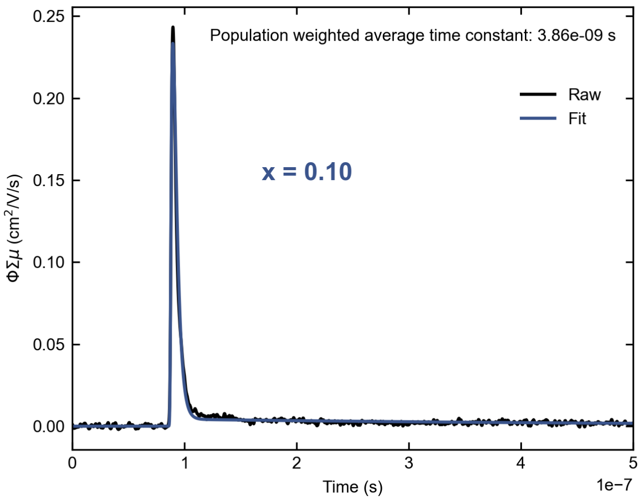

Figure S21: The plot shows raw (black line) and fitted (colored line) yield mobility product ($\phi\Sigma\mu$) for $MA_{1-x}en_xSn_{1-0.7x}I_{3-0.4x}$ $x = 0.10$.

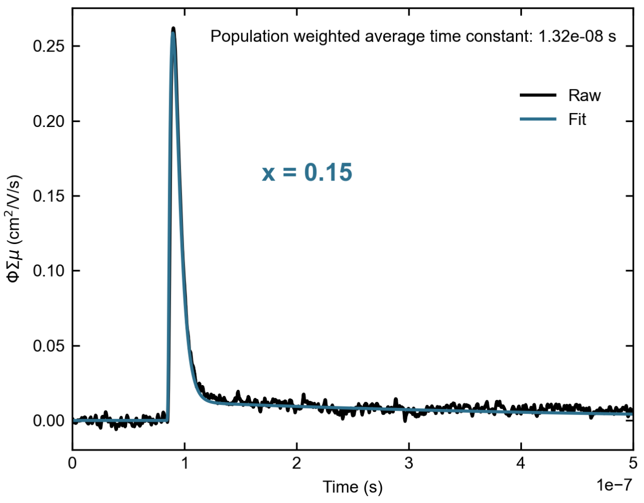

Figure S22: The plot shows raw (black line) and fitted (colored line) yield mobility product ($\phi\Sigma\mu$) for $MA_{1-x}en_xSn_{1-0.7x}I_{3-0.4x}$ x = 0.15.

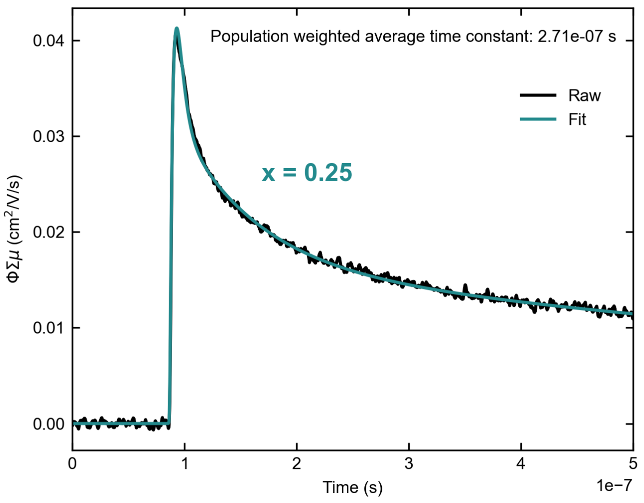

Figure S23: The plot shows raw (black line) and fitted (colored line) yield mobility product ($\phi\Sigma\mu$) for $MA_{1-x}en_xSn_{1-0.7x}I_{3-0.4x}$ where x = 0.25.

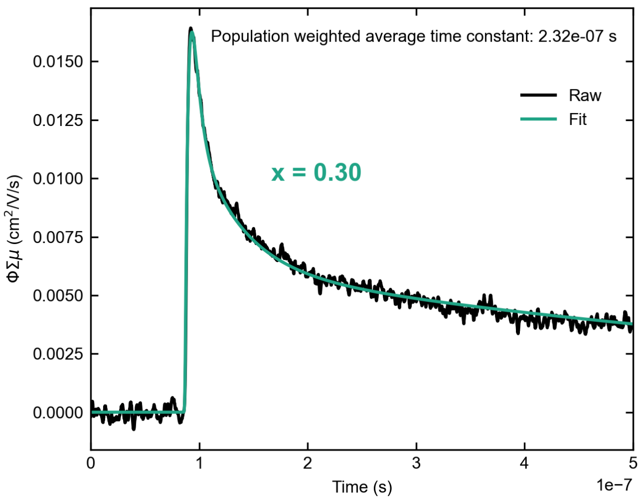

Figure S24: The plot shows raw (black line) and fitted (colored line) yield mobility product ($\phi\Sigma\mu$) for $MA_{1-x}en_xSn_{1-0.7x}I_{3-0.4x}$ x = 0.30.

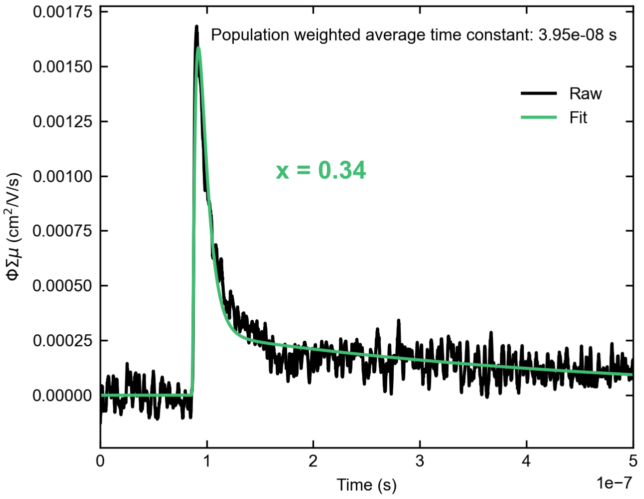

Figure S25: The plot shows raw (black line) and fitted (colored line) yield mobility product ($\phi\Sigma\mu$) for $MA_{1-x}en_xSn_{1-0.7x}I_{3-0.4x}$ x = 0.34.

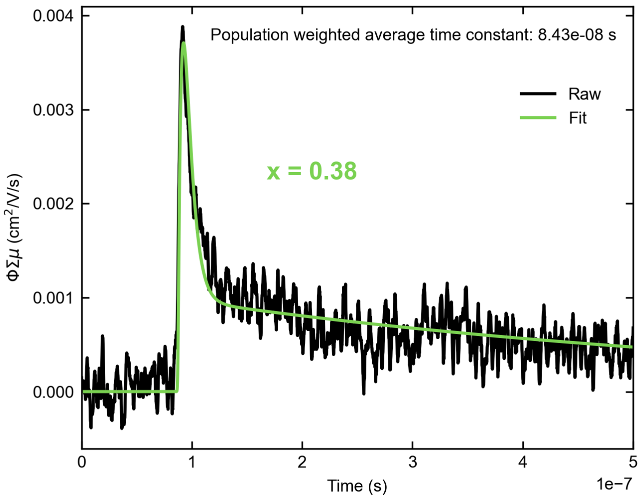

Figure S26: The plot shows raw (black line) and fitted (colored line) yield mobility product ($\phi\Sigma\mu$) for $MA_{1-x}en_xSn_{1-0.7x}I_{3-0.4x}$ x = 0.38.

References

(1) Spanopoulos, I.; Ke, W.; Stoumpos, C., Constantinos; Schueller, E. C.; Kontsevoi, O. Y.; Seshadri, R.; Kanatzidis, M. G. Unraveling the Chemical Nature of the 3D "Hollow" Hybrid Halide Perovskites. *J. Am. Chem. Soc.* **2018**, *140*, 5728–5742.

(2) Van Gompel, W. T. M.; Herckens, R.; Reekmans, G.; Ruttens, B.; D'Haen, J.; Adriaensens, P.; Lutsen, L.; Vanderzande, D. Degradation of the Formamidinium Cation and the Quantification of the Formamidinium–Methylammonium Ratio in Lead Iodide Hybrid Perovskites by Nuclear Magnetic Resonance Spectroscopy. *The Journal of Physical Chemistry C* **2018**, *122*, 4117–4124.

(3) Reid, O. G.; Moore, D. T.; Li, Z.; Zhao, D.; Yan, Y.; Zhu, K.; Rumbles, G. Quantitative analysis of time-resolved microwave conductivity data. *Journal of Physics D: Applied Physics* **2017**, *50*, 493002.

(4) Savenije, T. J.; Ferguson, A. J.; Kopidakis, N.; Rumbles, G. Revealing the Dynamics of Charge Carriers in Polymer: Fullerene Blends Using Photoinduced Time-Resolved Microwave Conductivity. *The Journal of Physical Chemistry C* **2013**, *117*, 24085–24103.

(5) Earley, J. D.; Zieleniewska, A.; Ripberger, H. H.; Shin, N. Y.; Lazorski, M. S.; Mast, Z. J.; Sayre, H. J.; McCusker, J. K.; Scholes, G. D.; Knowles, R. R.; Reid, O. G.; Rumbles, G. Ion-pair reorganization regulates reactivity in photoredox catalysts. *Nature Chemistry* **2022**, 1–8.

(6) Yuan, Z. et al. Discovery of the Zintl-phosphide $BaCd_2P_2$ as a long carrier lifetime and stable solar absorber. *Joule* **2024**, *8*, 1412–1429.
